# Global and national trends in years of life lost and years lived with disability caused by three common gastrointestinal cancers from 1990 to 2019

**DOI:** 10.1186/s12876-022-02567-5

**Published:** 2022-11-28

**Authors:** Danfeng Yu, Zejin Ou, Wenni Zhang, Huan He, Yongzhi Li, Wenqiao He, Minyi Zhang, Yuhan Gao, Fei Wu, Qing Chen

**Affiliations:** 1 Department of MICU, Guangdong Women and Children Hospital, Guangzhou, China; 2 Key Laboratory of Occupational Environment and Health, Guangzhou Twelfth People’s Hospital, Guangzhou, China; 3 Department of Epidemiology, Guangdong Provincial Key Laboratory of Tropical Disease Research, School of Public Health, Southern Medical University, 1838 Guangzhou North Road, Guangzhou, 510515 China

**Keywords:** Gastrointestinal cancer, Global burden of disease, Estimated annual percentage change, Years of life lost, Years lived with disability

## Abstract

**Background:**

Gastrointestinal cancers are a critical global cancer burden, and tracking their trends would inform the health policies.

**Methods:**

Trends of years of life lost (YLLs) and years lived with disability (YLDs) caused by three common gastrointestinal cancers were estimated using annual percentage change (EAPC) and age-standardized rate (ASR). Data was extracted from the Global Burden of Disease study 2019.

**Results:**

The ASR per 100,000 population-year of YLLs caused by esophageal cancer, stomach cancer, and colorectal cancer were 137.98, 264.15, and 282.51 in 2019, respectively. Their overall trends of YLLs declined during 1990–2019, with the respective EAPCs being − 1.42 (95% Confidence Interval [CI]: − 1.71 to − 1.13), − 2.13 (95%CI: − 2.29 to − 1.96), and − 0.25 (95%CI: − 0.30 to − 0.19). Meanwhile, decreasing trends of YLDs caused by esophageal cancer and stomach cancer were observed, in which the EAPCs were − 0.67 (95%: − 0.94 to − 0.40) and − 0.85 (95%CI: − 0.97 to − 0.73), respectively. However, an increasing trend was seen in that of colorectal cancer (EAPC = 0.83, 95%CI: 0.77 to 0.89). Among countries, the largest decrease in trend of YLLs was that of stomacher cancer in the Republic of Korea (EAPC =  − 5.88, 95%CI: − 6.07 to − 5.69). However, pronounced increasing trend of YLDs caused by colorectal cancer occurred in China (EAPC = 4.40, 95%CI: 4.07 to 4.72).

**Conclusions:**

Decreasing trends in YLLs and YLDs caused by esophageal cancer, stomach cancer, and colorectal cancer were observed in most countries and regions, indicating that the great progress had been achieved over the past decades. However, the cancer burden was geographical heterogeneity, and cost-effective measures were still required to decline the burden caused by gastrointestinal cancers.

**Supplementary Information:**

The online version contains supplementary material available at 10.1186/s12876-022-02567-5.

## Background

Gastrointestinal cancers are the leading causes of death globally. The survival patterns of gastrointestinal cancers had dramatically changed worldwide in recent decades [1, 2]. Years of life lost (YLLs) and years lived with disability (YLDs) are critical metrics of health loss, reflecting the socioeconomic status and development of the health care system. Therefore, tracking the trends of YLLs and YLDs caused by gastrointestinal cancers would provide updated information for public health strategies.

During 2006–2016, the age-standardized rate (ASR) of YLLs caused by stomach cancer, colorectal cancer, and esophageal cancer had changed with the estimated − 24.7%, − 8.9%, and − 22.0%, respectively [3]. In 2017, the number of disability adjusted life years (DALYs) caused by colorectal cancer was 19.0 million, comprising 95% from YLLs and 5% from YLDs; that of stomach cancer was 19.1 million, with 98% from YLLs and 2% from YLDs; that of esophageal cancer was 9.78 million, comprising 98.7% from YLLs sand 1.3% from YLDs globally [4]. The clinical outcomes of gastrointestinal cancers were mainly promoted by the progress in screening, treatment, and management [5]. For example, population-based early screening programs have been available since 1990s, and approximately 65% of US and 52% of Japan adults participated the colorectal cancer screening, which gained dramatical progress in survival [6]. Meanwhile, China government had implemented targeted policies and investments to improve health care systems, and achieved decreasing trends in age-standardized mortality rates of stomach and esophagus cancers [7, 8]. Meanwhile, and the standardized rate of DALYs caused by gastric cancer declined in recent years [9], e.g. the age‐standardized DALYs rates of esophageal cancer declined by 45.3% from 1990 to 2019 [10]. However, the burden of gastrointestinal cancers exists significant geographic heterogeneity, and more detailed data on the trends in different countries is necessary to be tracked.

Estimated annual percentage changes (EAPC) is widely used in public health research, which can be used to assess the temporal changes in ASR in a time interval [11]. In order to provide updated epidemiological trends of the common gastrointestinal cancers, this work adopted EAPC to estimate spatial and temporal trends in the ASRs of YLLs and YLDs caused by gastrointestinal cancers worldwide from 1990 to 2019.

## Material and methods

### Data source

According to the GBD, YLLs is the years of life lost due to premature death. YLDs is a value that represents average lifespan of incident cases till rehabilitation or death, and disability weight due to that status. Data on YLLs and YLDs caused by three common gastrointestinal cancers, including esophageal cancer, stomach cancer, and colorectal cancer, were collected from the Global Health Data Exchange (GHDx) query tool (http://ghdx.healthdata.org/gbd-results-tool). Data was extracted in sexes, 21 geographic regions, and 204 countries/territories during the period 1990–2019. According to the index of socio-demographic index (SDI), these regions and countries were divided into five levels by: high, high-middle, middle, low-middle, and low. The Human Development Index (HDI) reflects the conditions and availability of health resources, which was downloaded from the United Nations Development Program (http://hdr.undp.org/en/data).

### Statistical analysis

ASR is an indispensable index when comparing the discrepancies in the age structure of different populations. The ASR per 100,000 population-year is calculated as follows:

$$\mathbf{ASR} = \frac{\boldsymbol{\Sigma}\boldsymbol{i}^{\boldsymbol{A}}_{\boldsymbol{=1}} \boldsymbol{a}_{\boldsymbol{i}} \boldsymbol{w}_{i}}{\boldsymbol{\Sigma}\boldsymbol{i}^{\boldsymbol{A}}_{\boldsymbol{=1}}\boldsymbol{w}_{i}} \times \boldsymbol{100,\!000}$$

In the formula, *ai* means the age-specific rate in the *i*th age group, *wi* means the number of persons (or the weight) in the corresponding *i*th age subgroup of the selected reference standard population, and A is the number of age groups.

When describing the magnitude of trends in ASR, EAPC is regarded as a widely used method [12–14]. A regression line is matched to the natural logarithm of the rates (ASR). And then the linear regression model was used to calculated the EAPC and its 95% confidence interval (CI). The formulas were as follows:

$$\mathbf y\boldsymbol=\mathbf\alpha\boldsymbol+\mathbf\beta{x}\boldsymbol+\mathbf\varepsilon$$

$$\mathbf{EAPC}\boldsymbol=\mathbf{100}\boldsymbol\times\boldsymbol(\mathbf{exp}\boldsymbol(\mathbf\beta\boldsymbol)\boldsymbol-\mathbf1\boldsymbol)$$

in which y equals $\mathrm{ln}(ASR)$, and x is the corresponding year. The trends were determined as follows: (1). both EAPC value and its 95%CI > 0 represented an increasing trend; (2). both EAPC value and its 95%CI < 0 represented a decreasing trend; (3) other outcomes signified that ASR was relatively stable. With the aim of exploring on the influential factors of EAPC, the correlations between EAPCs and ASR in 1990, and between EAPCs and HDI in 2019 were estimated using Pearson correlation analysis. Data were analyzed using R 3.6.2 (Lucent Technologies, Jasmine Mountain, USA). A *p* value of < 0.05 was deemed to be statistically significant.

## Results

### Trends in YLLs and YLDs caused by esophageal cancer

**Fig. 1:**
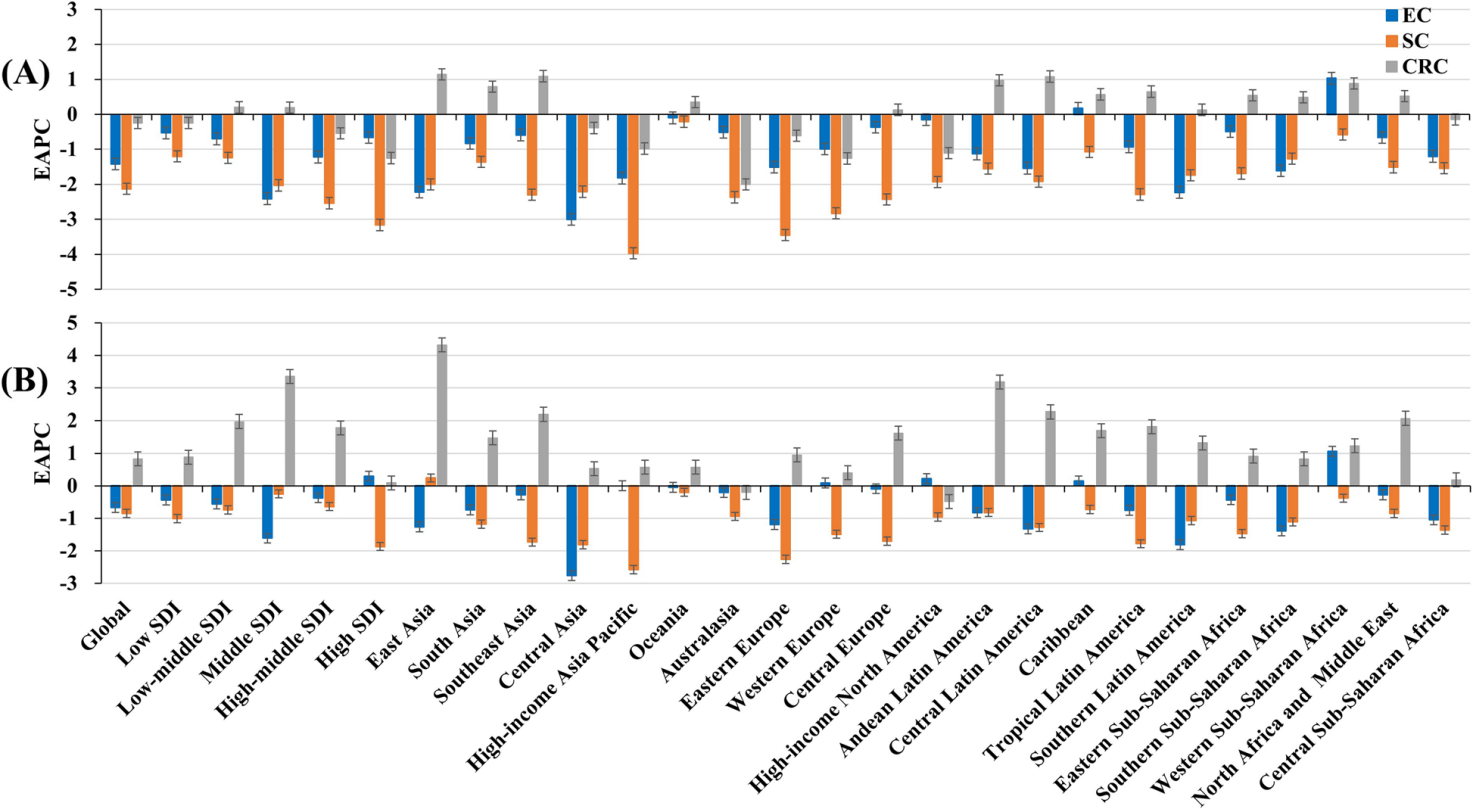
Trends of YLLs and YLDs caused by gastrointestinal cancers globally 1990–2019. (**A**) and (**B**) were the EAPCs of YLLs and YLDs, respectively. YLLs: years of life lost; YLDs: years lived with disability; EAPC: Estimated annual percentage change; SDI: sociodemographic index

**Fig. 2:**
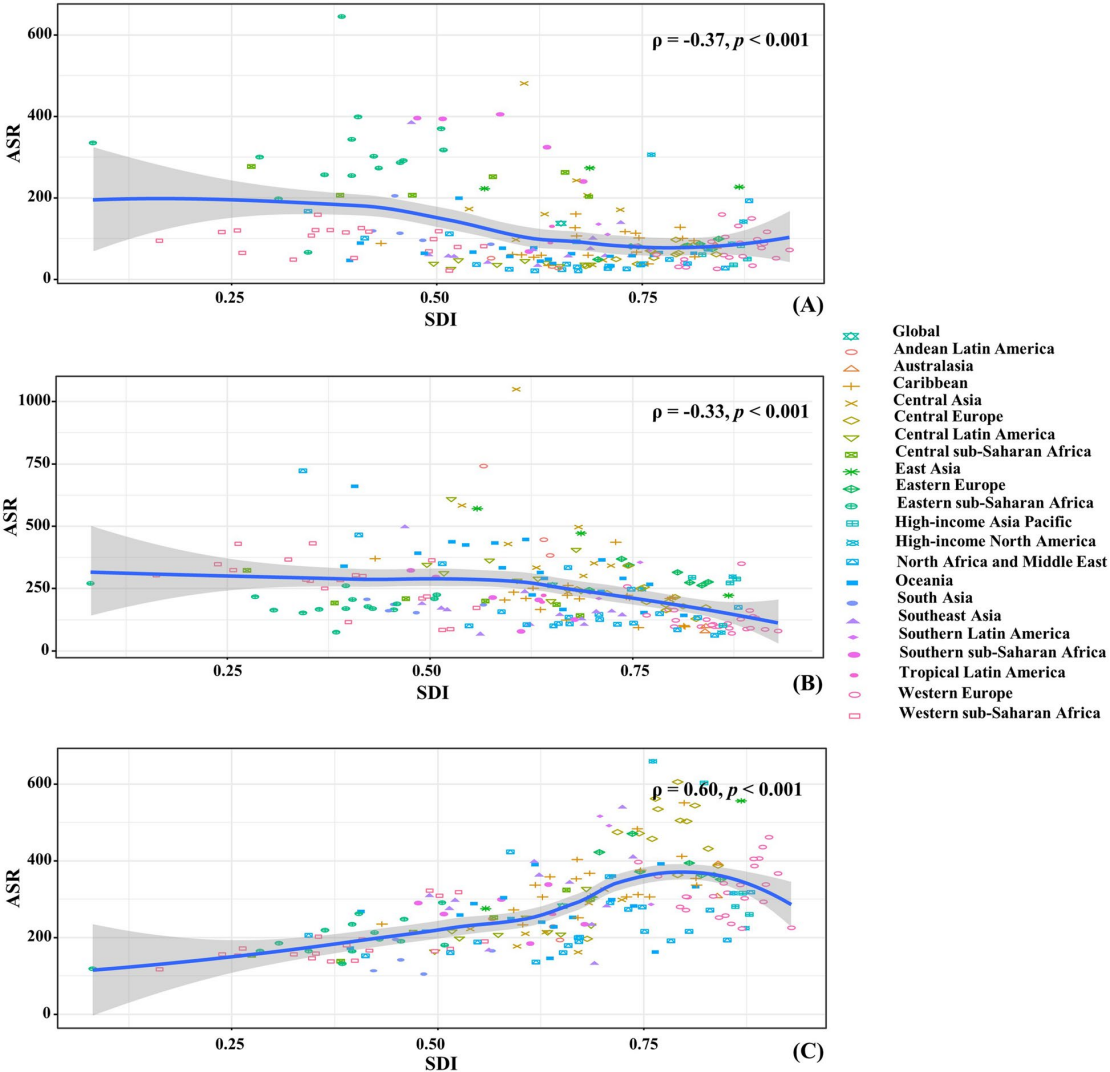
The associations between ASRs of gastrointestinal cancers and SDI in 2019 among regions. (**A**), (**B**) and (**C**) were ASRs of YLLs caused by esophageal cancer, stomach cancer, and colorectal cancer, respectively. The association was calculated with Pearson correlation analysis. The symbols were the countries/territories in the corresponding regions. ASR, age-standardized rate; socio-demographic index Age-standardized rates; YLLs: years of life lost

**Fig. 3:**
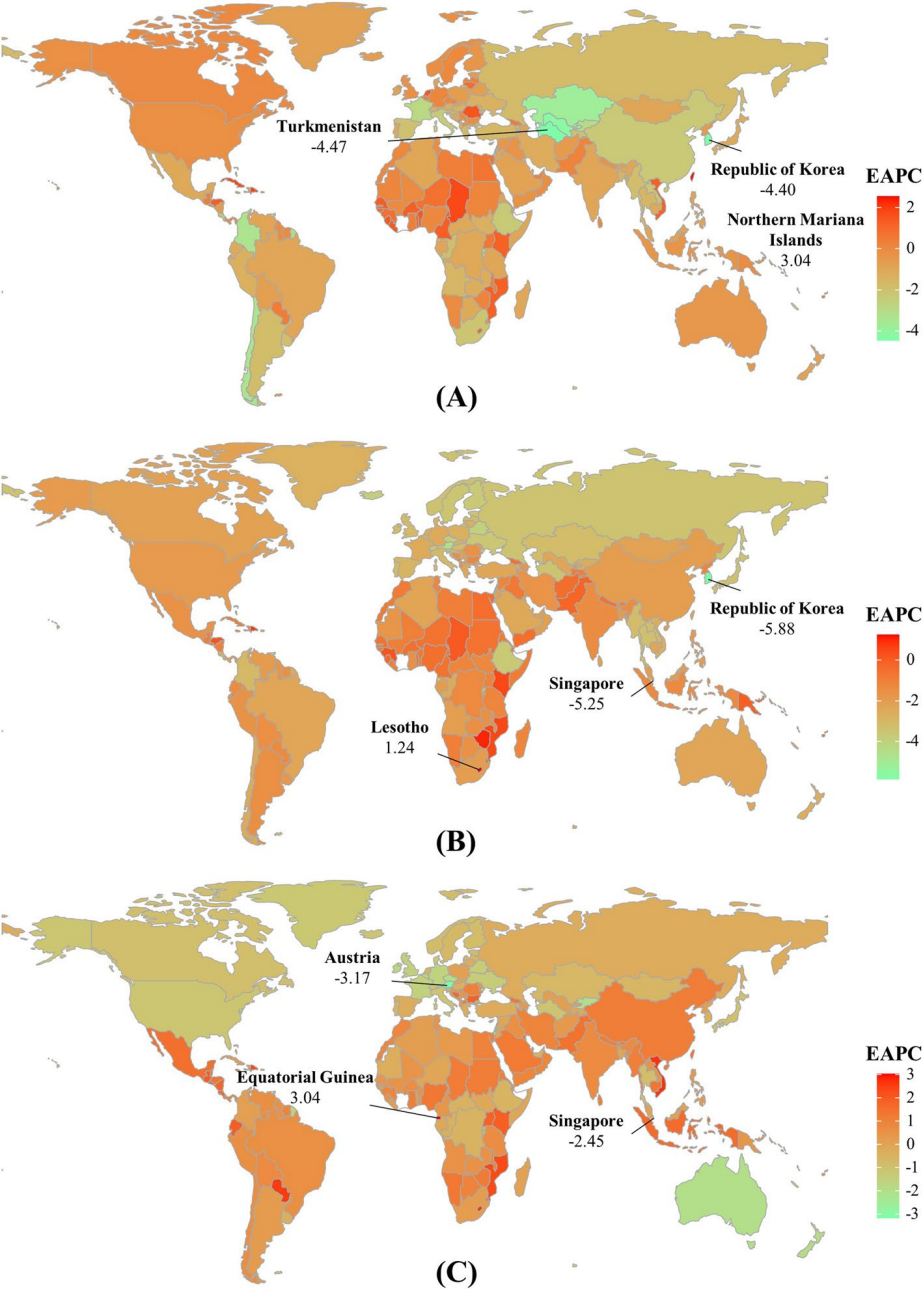
Trends of YLLs caused by gastrointestinal cancers at the national level from 1990 to 2019. (**A**), (**B**), and (**C**) presented the EAPCs of esophageal cancer, stomach cancer, and colorectal cancer worldwide, respectively. Countries/territories with an extreme value were annotated. YLLs: years of life lost; EAPC, estimated annual percentage change

Globally, the number of YLLs caused by esophageal cancer was 11.52 (95% uncertainty interval [UI]: 10.24 to 12.79) million in 2019, with an increase of 41.77% since 1990. The overall ASR of YLLs decreased with an annual average of 1.42% from 1990 to 2019 (EAPC: − 1.42, 95% CI: − 1.71 to − 1.13). Decreasing trends of YLLs were seen in both sexes, particularly Female (EAPC =  − 2.32, 95%CI: − 2.65 to − 1.99). Decreasing trends were observed in all SDI areas, particularly middle one (EAPC =  − 2.41, 95%CI: − 2.85 to − 1.98). In 2019, the ASRs of YLLs ranged from 33.02/100,000 in Andean Latin America to 271.73/100,000 in East Asia. Decreasing trends of YLLs were observed in 20 regions, especially Central Asia and East Asia, with the EAPCs were − 3.00 (95% CI: − 3.23 to − 2.77) and − 2.22 (95% CI: − 2.70 to − 1.74), respectively. Whereas increasing trend occurred in Western Sub-Saharan Africa (EAPC = 1.03, 95%CI: 0.92 to 1.15) (Table 1; Fig. 1A, and Supplementary Fig. 1A-B). A negative association was found between trends of YLLs and SDI among regions (*ρ* =  − 0.37, *p* < 0.001; Fig. 2A). At the national level, the highest increasing percent of YLLs number occurred in United Arab Emirates (1128.46%) and Qatar (448.83%). Decreasing trends in ASRs of YLLs were observed in 136 countries/territories from 1990 to 2019, with the largest increase occurring in Turkmenistan (EAPC =  − 4.47, 95%CI: − 5.22 to − 3.72), followed by Republic of Korea and Singapore. Conversely, increasing trends were observed in forty-five countries, and the most pronounced ones occurred in Northern Mariana Islands and Taiwan (China), with the respective EAPCs were 3.04 (95%CI: 2.60 to 3.48) and 2.56 (95%CI: 2.29 to 2.82) (Supplementary table 3; Fig. 3A, and Supplementary Fig. 5A).

**Table 1:** The percentage changes in number and EAPCs of YLLs and YLDs due to esophageal cancer from 1990 to 2019 in global, sexes, SDI areas and geographic regions

**Characteristics**	**YLLs**				**YLDs**			
	2019		1990 − 2019		2019		1990–2019	
	Number × 10^3^ (95% UI)	ASR/100,000 (95% UI)	Percentage change (%)	EAPC (95%CI)	Number × 10^3^ (95% UI)	ASR/100,000 (95% UI)	Percentage change (%)	EAPC (95%CI)
**Overall**	11,515.94 (10,243.45–12,787.77)	137.98 (122.84–153.13)	41.77	− 1.42 (− 1.71- − 1.13)	150.07 (107.07–195.66)	1.82 (1.30–2.37)	76.37	− 0.67 (− 0.94- − 0.40)
**Sex**								
Male	8714.07 (7522.78–9973.75)	218.61 (188.83–249.68)	53.47	− 1.09 (− 1.37- − 0.81)	107.65 (76.29–142.65)	2.77 (1.97–3.66)	86.89	− 0.41 (− 0.67- − 0.16)
Female	2801.88 (2391.56–3157.41)	64.32 (54.90–72.49)	14.58	− 2.32 (− 2.65- − 1.99)	42.43 (29.24–56.39)	0.97 (0.67–1.29)	54.34	− 1.28 (− 1.59- − 0.97)
**SDI**								
Low	797.97 (655.60–961.45)	139.67 (115.41–167.23)	93.06	− 0.53 (− 0.59- − 0.47)	7.57 (5.10–10.70)	1.41 (0.95–1.98)	96.78	− 0.44 (− 0.49- − 0.39)
Low-middle	1595.29 (1417.74–2230.25)	110.09 (97.94–154.26)	86.57	− 0.70 (− 0.79- − 0.62)	16.37 (11.42–23.58)	1.18 (0.82–1.69)	98.88	− 0.56 (− 0.63- − 0.48)
Middle	4429.46 (3685.98–5144.02)	172.91 (143.68–199.99)	25.15	− 2.41 (− 2.85- − 1.98)	56.19 (38.52–75.15)	2.26 (1.54–3.03)	59.69	− 1.61 (− 2.00- − 1.21)
High-middle	3065.32 (2451.81–3550.22)	149.07 (119.40–172.55)	38.54	− 1.22 (− 1.52- − 0.91)	40.28 (27.24–54.66)	1.96 (1.32–2.65)	77.08	− 0.37 (− 0.62- − 0.11)
High	1624.35 (1542.69–1699.85)	94.18 (89.90–98.55)	47.52	− 0.66 (− 0.74- − 0.58)	29.62 (21.31–38.25)	1.64 (1.18–2.11)	96.72	0.30 (0.12–0.48)
**Regions**								
East Asia	5844.62 (4655.17–7079.84)	271.73 (218.22–327.93)	29.37	− 2.22 (− 2.70- − 1.74)	78.25 (53.50–106.31)	3.71 (2.53–5.01)	73.05	− 1.26 (− 1.68- − 0.83)
South Asia	1461.86 (1271.16–1942.4)	97.27 (84.60–129.94)	98.23	− 0.83 (− 0.95- − 0.72)	14.73 (10.08–20.58)	1.02 (0.70–1.43)	110.27	− 0.74 (− 0.85- − 0.63)
Southeast Asia	399.27 (339.14–466.89)	61.01 (52.14–71.63)	100.53	− 0.59 (− 0.64- − 0.54)	4.45 (3.02–6.07)	0.71 (0.48–0.97)	122.56	− 0.28 (− 0.32- − 0.24)
Central Asia	128.52 (113.09–152.20)	163.06 (144.16–191.08)	− 24.02	− 3.00 (− 3.23- − 2.77)	1.29 (0.90–1.76)	1.74 (1.22–2.36)	− 21.71	− 2.76 (− 2.99- − 2.53)
High-income Asia Pacific	297.17 (273.88–323.42)	73.66 (68.74–80.33)	24.00	− 1.82 (− 1.94- − 1.7)	8.95 (6.30–12.19)	2.10 (1.47–2.88)	112.97	0 (− 0.23–0.23)
Oceania	4.17 (3.10–5.59)	52.99 (39.94–70.95)	130.51	− 0.10 (− 0.14- − 0.07)	0.04 (0.03–0.06)	0.59 (0.36–0.89)	133.07	− 0.05 (− 0.08- − 0.03)
Australasia	39.26 (35.94–42.64)	83.91 (77.07–91.15)	79.21	− 0.51 (− 0.57- − 0.45)	0.62 (0.41–0.87)	1.27 (0.85–1.79)	105.66	− 0.21 (− 0.32- − 0.09)
Eastern Europe	274.45 (238.22–313.34)	82.57 (71.71–94.21)	− 13.59	− 1.51 (− 1.76- − 1.26)	3.09 (2.12–4.15)	0.91 (0.63–1.23)	− 5.84	− 1.19 (− 1.44- − 0.94)
Western Europe	694.77 (657.35–728.06)	87.21 (82.93–91.38)	17.01	− 0.99 (− 1.07- − 0.91)	12.04 (8.46–16)	1.44 (1.01–1.91)	59.71	0.09 (− 0.04–0.23)
Central Europe	142.08 (123.15–162.11)	73.89 (63.80–84.54)	24.4	− 0.37 (− 0.49- − 0.25)	1.63 (1.12–2.17)	0.81 (0.56–1.09)	37.65	− 0.09 (− 0.19–0.01)
High-income North America	517.25 (496.69–535.63)	87.09 (83.86–90.13)	78.97	− 0.15 (− 0.24- − 0.06)	7.38 (5.02–9.87)	1.21 (0.82–1.62)	99.90	0.22 (0.09–0.36)
Andean Latin America	18.61 (14.92–23.38)	33.02 (26.52–41.49)	90.23	− 1.13 (− 1.22- − 1.03)	0.23 (0.15–0.32)	0.41 (0.27–0.58)	114.56	− 0.83 (− 0.92- − 0.74)
Central Latin America	89.69 (75.74–105.87)	37.50 (31.72–44.25)	87.94	− 1.54 (− 1.66- − 1.42)	1.09 (0.74–1.48)	0.46 (0.32–0.63)	108.23	− 1.32 (− 1.43- − 1.22)
Caribbean	46.79 (39.66–54.05)	89.69 (76.00–103.48)	94.72	0.17 (− 0.03–0.37)	0.53 (0.37–0.72)	1.02 (0.7–1.38)	98.74	0.15 (− 0.03–0.33)
Tropical Latin America	324.99 (308.54–341.52)	129.64 (123.04–136.29)	92.93	− 0.93 (− 0.99- − 0.87)	3.44 (2.43–4.44)	1.39 (0.98–1.80)	109.84	− 0.75 (− 0.81- − 0.70)
Southern Latin America	82.16 (76.60–88.02)	99.83 (93.20–106.84)	3.27	− 2.23 (− 2.38- − 2.07)	1.05 (0.69–1.48)	1.25 (0.82–1.78)	18.72	− 1.81 (− 1.97- − 1.65)
Eastern Sub-Saharan Africa	472.4 (359.07–602.64)	260.86 (198.62–329.79)	94.61	− 0.49 (− 0.57- − 0.41)	4.35 (2.82–6.52)	2.57 (1.68–3.82)	95.57	− 0.43 (− 0.50- − 0.36)
Southern Sub-Saharan Africa	158.34 (141.23–186.50)	264.41 (236.57–307.53)	49.39	− 1.61 (− 2.16- − 1.05)	1.54 (1.09–2.07)	2.70 (1.92–3.59)	57.32	− 1.39 (− 1.90- − 0.89)
Western Sub-Saharan Africa	136.56 (104.06–165.64)	67.11 (51.13–80.71)	170.42	1.03 (0.92–1.15)	1.36 (0.89–1.87)	0.72 (0.47–0.98)	167.72	1.06 (0.95–1.17)
North Africa and Middle East	256.66 (181.01–298.51)	54.93 (39.98–63.11)	111.84	− 0.66 (− 0.71- − 0.61)	2.83 (1.86–3.79)	0.65 (0.43–0.86)	133.58	− 0.28 (− 0.32- − 0.23)
Central Sub-Saharan Africa	126.33 (67.90–171.07)	213.06 (114.67–290.28)	75.93	− 1.20 (− 1.3- − 1.11)	1.18 (0.59–1.81)	2.16 (1.07–3.30)	80.75	− 1.05 (− 1.13- − 0.96)

*YLLs* years of life lost, *YLDs* years lived with disability, *EAPC* estimated annual percentage change, *ASR* age-standardized rate, *CI* confidence interval, *UI* uncertainty interval, *SDI* socio-demographic index

**Fig. 4:**
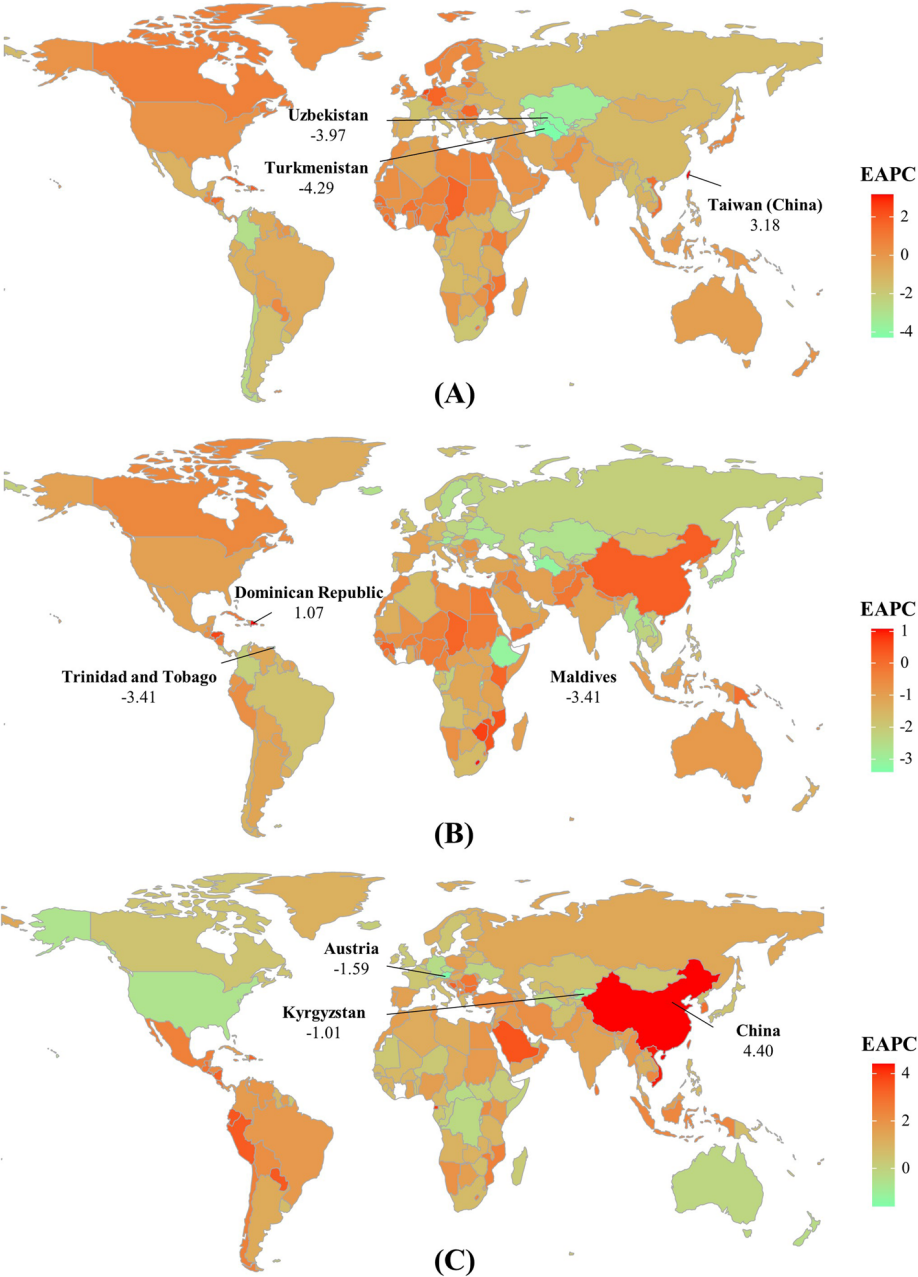
Trends of YLDs caused by gastrointestinal cancers at the national level from 1990 to 2019. (**A**), (**B**), and (**C**) presented the EAPCs of esophageal cancer, stomach cancer, and colorectal cancer worldwide, respectively. Countries/territories with an extreme value were annotated. YLDs: years lived with disability; EAPC, estimated annual percentage change

The number of YLDs caused by esophageal cancer was 0.15 (95% UI: (0.11 to 0.20) million worldwide in 2019, with an increase of 76.37% from 1990. Decreasing trend in ASR of YLDs was observed globally from 1990 to 2019 (EAPC =  − 0.67, 95%CI: − 0.94 to − 0.40). The larger decreasing trend was seen in the female patients (EAPC =  − 1.28, 95%CI: − 1.59 to − 0.97). Decreasing trends of YLDs were observed in most SDI areas, particularly the middle one (EAPC =  − 1.61, 95%CI: − 2.00 to − 1.21). The ASRs of YLDs varied from 0.41/100,000 in Andean Latin America to 3.71/100,000 in East Asia in 2019. Decreasing trends were seen in fifteen regions, particularly Central Asia (EAPC =  − 2.76, 95%CI: − 2.99 to − 2.53). Whereas increasing trends occurred in Western Sub-Saharan Africa and High-income North America (Table 1; Fig. 1B, and Supplementary Fig. 1C-D). A negative association was demonstrated between trends of YLDs and SDI among regions (*ρ* =  − 0.24, *p* < 0.001; Supplementary Fig. 4A). Among 204 countries/territories, the highest increasing percent of number occurred in the United Arab Emirates (1132.05%) and Qatar (546.61%). Decreasing trends of YLDs were found in 114 settings from 1990 to 2019, particularly Turkmenistan and Uzbekistan, with the respective EAPCs being − 4.29 (95%CI: − 5.01 to − 3.55) and − 3.97 (95%CI: − 4.41 to − 3.52). On the other hand, increasing trends were observed in sixty-seven ones, and the largest increasing in Taiwan (China) (EAPC = 3.18, 95CI: 2.88 to 3.48) (Supplementary table 4; Fig. 4A, and Supplementary Fig. 6A).

### Trends in YLLs and YLDs caused by stomach cancer

The number of YLLs caused by stomach cancer was 21.87 (95% UI: 19.97 to 23.71) million globally in 2019, with an increase of 8.06% since 1990. Trend in the overall ASRs of YLLs declined from 1990 to 2019 (EAPC =  − 2.13, 95% CI: − 2.29 to − 1.96). Compared with males, a more pronounced decreasing trend was seen in female patients. Decreasing trends of YLLs were observed in all SDI areas, particularly the high one (EAPC: − 3.16, 95% CI: − 3.22 to − 3.10). The ASRs of YLLs varied from 75.49/100,000 in High-income North America to 469.50/100,000 in East Asia in 2019. Decreasing trends of YLLs were observed in all geographic regions, particularly High-income Asia Pacific (EAPC =  − 3.97, 95%CI: − 4.04 to − 3.89) (Supplementary table 1; Fig. 1A, and Supplementary Fig. 2A-B). A negative association was found between trends of YLLs and SDI among regions (*ρ* =  − 0.33, *p* < 0.001; Fig. 2B). Among 204 countries/territories, the highest increasing percent of number occurred in United Arab Emirates (480.44%) and Qatar (260.09%), while the largest decrease was seen in Latvia (− 57.78%). Decreasing trends of YLLs were observed in 193 countries/territories, and the largest decreasing ones were seen in Republic of Korea and Singapore, in which the respective EAPCs were − 5.88 (95%CI: − 6.07 to − 5.69) and − 5.25(95%CI: − 5.45 to − 5.06). However, increasing trends were seen in seven countries, including Lesotho, Zimbabwe, and Dominican Republic (Supplementary table 3; Fig. 3B, and Supplementary Fig. 5B).

Globally, the number of YLDs caused by stomach cancer was 0.35 (95%UI: 0.25 to 0.46) million in 2019, with an increase of 57.47% from 1990. Decreasing trend in ASRs of YLDs was seen worldwide from 1990 to 2019 (EAPC: − 0.85, 95% CI: − 0.97 to − 0.73). Compared with males, a larger decreasing trend was found in female patients. Decreasing trends of YLDs were seen in all SDI areas, and the largest decrease occurred in the high SDI areas (EAPC =  − 1.87, 95%CI: − 1.95 to − 1.80). The ASRs of YLDs varied from 1.55/100,000 in Southern Sub-Saharan Africa to 9.09/100,000 in High-income Asia Pacific in 2019. Decreasing trends of YLDs were observed in all geographic regions, particularly high-income Asia Pacific and Eastern Europe, in which the respective EAPCs were − 2.58 (95%CI: − 2.67 to − 2.48) and − 2.26 (95%CI: − 2.45 to − 2.07) (Supplementary table 1; Fig. 1B, and Supplementary Fig. 2C-D). At the national level, the highest increasing percent of number occurred in the United Arab Emirates (519.22%) and Qatar (438.91%). Decreasing trends in ASRs of YLDs occurred in 186 countries/territories, particularly Trinidad and Tobago (EAPC =  − 3.41, 95%CI: − 3.71 to − 3.11), followed by Maldives and Turkmenistan. Whereas increasing trends were seen in eight countries, including Dominican Republic and Cyprus, with the respective EAPCs were 1.07 (95%CI: 0.92 to 1.22) and 1.03 (95%CI: 0.72 to 1.35) (Supplementary table 4; Fig. 4B, and Supplementary Fig. 6B).

### Trends in YLLs and YLDs caused by colorectal cancer

The global number of YLLs caused by colorectal cancer increased 93.28% from 1990, and was 23.22 (95% UI: 21.66 to 24.59) million in 2019. The overall ASR of YLLs showed decreasing trend from 1990 to 2019 (EAPC =  − 0.25, 95%CI: − 0.30 to − 0.19). Male patients had higher number of YLLs, and increasing trend of YLLs. The largest decreasing trend was observed in the high SDI area (EAPC =  − 1.25, 95%CI: − 1.31 to − 1.19), while increasing in other two SDI areas. Among 21 geographic regions, the ASR of YLLs ranged from 161.95/100,000 in South Asia to 494.53/100,000 in Central Europe in 2019. decreasing trends of YLLs were seen in six regions, and the largest decreases occurred in Australasia (EAPC =  − 2.00, 95%CI: − 2.16 to − 1.84). Whereas increasing trends were found in eight regions (Supplementary table 2; Fig. 1A, and Supplementary Fig. 3A-B). A negative association was found between ASRs of YLLs and SDI among regions in 2019 (*ρ* = 0.60, *p* < 0.001; Fig. 2C). At the national level, the highest increasing percent of number occurred in United Arab Emirates (767.53%) and Qatar (629.87%), while the largest decrease was seen in Austria (− 31.37%). Decreasing trends of YLLs were observed in sixty-nine countries/territories, particularly Austria and Singapore, with the respective EAPCs of − 3.17 (95%CI: − 3.33 to − 3.01) and − 2.45 (95%CI: − 2.57 to − 2.34). On the other hand, increasing trends were observed in 118 ones, and the most pronounced increases occurred in Equatorial Guinea (EAPC = 3.04, 95%CI: 2.78 to 3.30), followed by Viet Nam and Lesotho (Supplementary table 3; Fig. 3C, and Supplementary Fig. 5C).

Globally, the number of YLDs caused by colorectal cancer was 1.07 (95% UI: 0.78 to 1.38) million in 2019; an increase of 169.70% from 1990. Increasing trend of YLDs were observed worldwide from 1990 to 2019 (EAPC = 0.83, 95%CI: 0.77 to 0.89). Increasing trends of YLDs were seen in both sexes, and male had more pronounced increase (EAPC = 1.21, 95%CI: 1.14 to 1.28). Upward trends were seen in most SDI areas, and the largest increase was in the middle one (EAPC = 3.35, 95%CI: 3.18–3.53). At the regional level, the ASR of YLDs ranged from 3.10/100,000 in Western Sub-Saharan Africa to 26.84/100,000 in Australasia in 2019. Increasing trends of YLDs were seen in eighteen regions, particularly East Asia (EAPC = 4.32, 95%CI: 4.01 to 4.62). Whereas decreasing trends only occurred in High-income North America and Australasia (Supplementary table 2; Fig. 1B, and Supplementary Fig. 3C-D). A negative association was demonstrated between ASRs of YLDs and SDI among regions in 2019 (*ρ* = 0.76, *p* < 0.001; Supplementary Fig. 4B). Among 204 countries/territories, the highest increasing percent of number occurred in Qatar (1365.26%) and the United Arab Emirates (1070.57%). Increasing trends of YLDs were found in 176 countries, and the largest increases occurred in China (EAPC = 4.40, 95%CI: 4.07 to 4.72), followed by Viet Nam and Equatorial Guinea. Whereas decreasing trends were seen in eleven countries, including Austria (EAPC =  − 1.59, 95%CI: − 1.76 to − 1.42), Kyrgyzstan, and United States of America (Supplementary table 4; Fig. 4C, and Supplementary Fig. 6C).

### Analysis of the influential factors of EAPCs

**Fig. 5:**
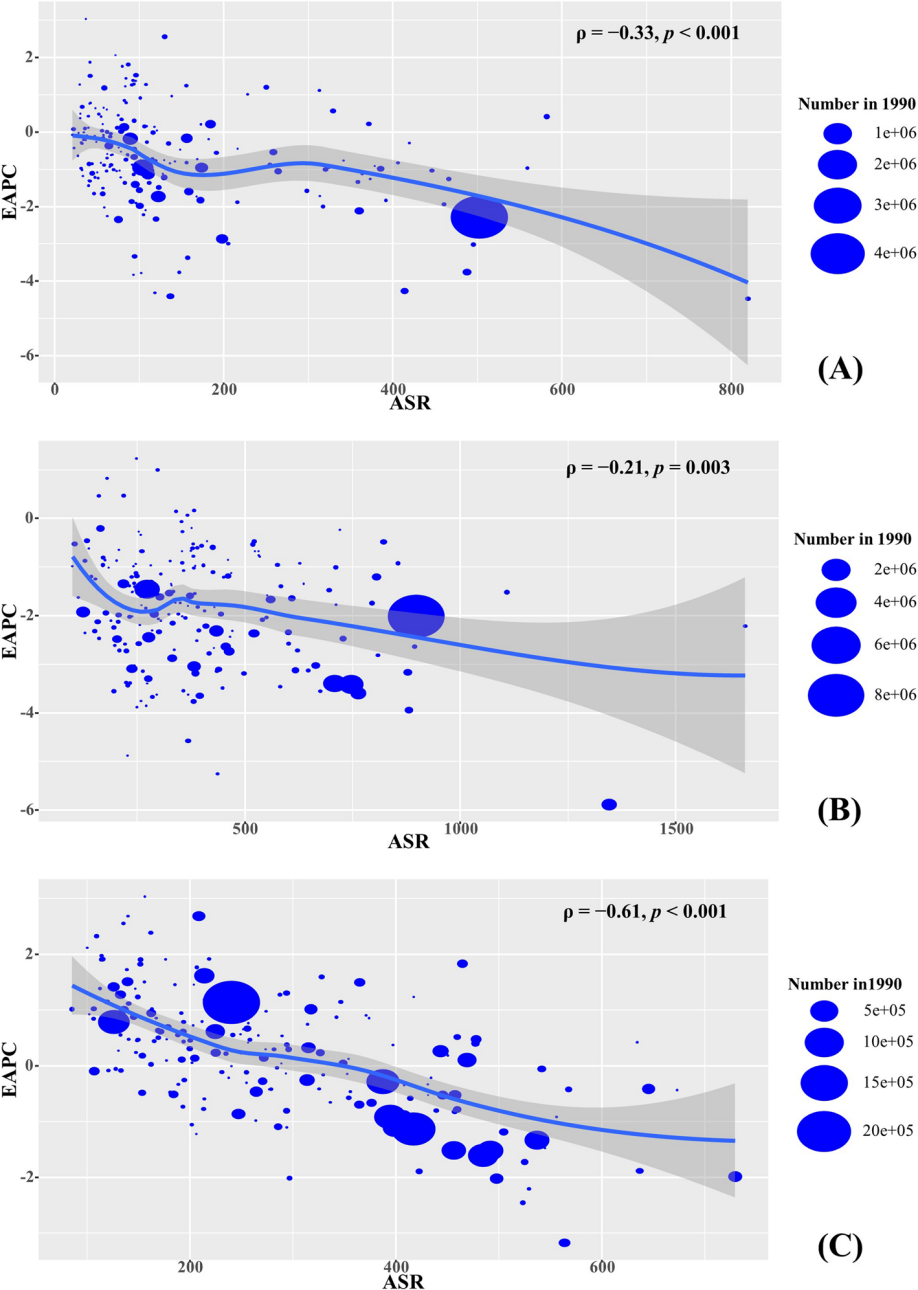
The association between EAPCs of YLLs and ASRs in 1990 at the national level. The EAPCs of YLLs caused by esophageal cancer (**A**), stomach cancer (**B**), and colorectal cancer (**C**) had negative associations with the corresponding ASR in 1990. The association was calculated with Pearson correlation analysis. The size of circle is increased with the numbers of YLLs in 1990. YLLs: years of life lost; EAPC, estimated annual percentage change

**Fig. 6:**
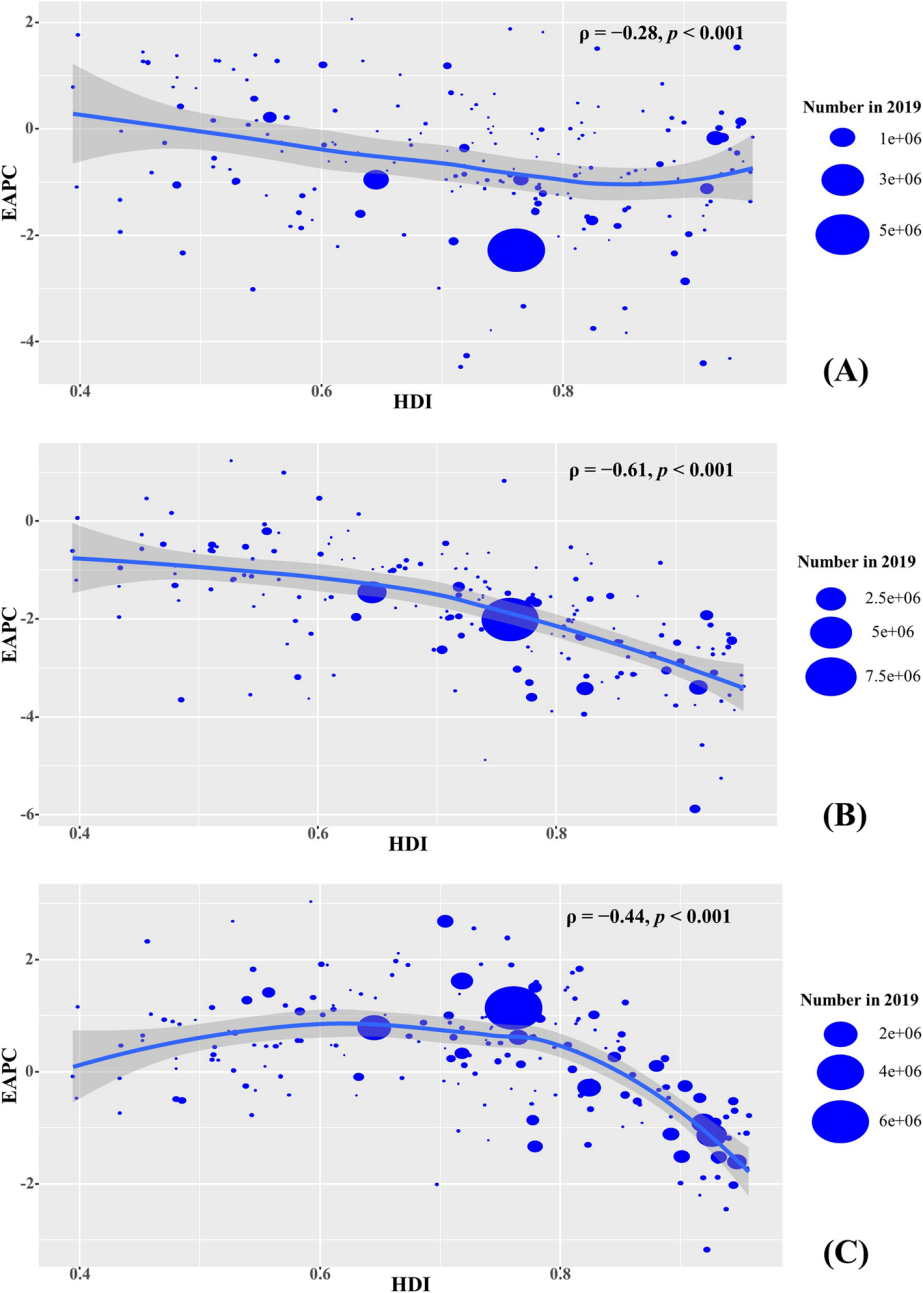
The association between EAPCs of YLLs and HDI in 2019 at the national level. The EAPCs of YLLs due to esophageal cancer (**A**), stomach cancer (**B**), and colorectal cancer (**C**) had negative associations with the HDI in 2019. The association was calculated with Pearson correlation analysis. The size of circle is increased with the numbers of YLLs in 2019. YLLs: years of life lost; EAPC, estimated annual percentage change; HDI, human development index

Negative associations were observed between EAPCs of YLLs caused by esophageal cancer, stomach cancer, and colorectal cancer and the ASR in 1990 (*ρ* =  − 0.33, *p* < 0.001; *ρ* =  − 0.21, *p* = 0.003; *ρ* =  − 0.61, *p* < 0.001; Fig. 5A-C, respectively). Meanwhile, negative associations also were found between EAPCs of YLLs caused by the above cancers and the HDI in 2019 (*ρ* =  − 0.28, *p* < 0.001; *ρ* =  − 0.61, *p* < 0.001; *ρ* =  − 0.44, *p* < 0.001, Fig. 6A-C, respectively).

In terms of YLDs, their EAPCs of esophageal cancer, stomach cancer, and colorectal cancer were negatively associated with the ASR in 1990 (*ρ* =  − 0.40, *p* < 0.001; *ρ* =  − 0.29, *p* < 0.001; *ρ* =  − 0.32, *p* < 0.001, Supplementary Fig. 7A-C, respectively), but not with the HDI in 2019.

## Discussion

In the present article, YLLs generally contributed to more than 90% of DALYs, and had more pronounced decreasing trends than YLDs, indicating the progresses in prevention, and early detection, and treatment [15]. Decreasing trends in ASRs of YLLs and YLDs caused by esophageal cancer and stomach cancer were observed worldwide, which was similar to the findings of the GBDs 2017 [16]. Particularly stomach cancer presented the largest decreasing trends of YLLs, which explained by the survival rates of stomach cancer had generally improved over the past decades, with about a 20% increase in the 5-year survival rate [17]. The results showed that progress in YLLs and YLDs of gastrointestinal cancers, which probably owed to the implementation of effective health promotion programs, and health-related investments in many countries under the frame of Sustainable development Goals [18, 19]. The substantial gains from the progress in treatments and detection of gastrointestinal cancers [20], especially the population-based endoscopic screening have been widely implemented to detect gastrointestinal cancers at early stages in recent years [21, 22]. For example, early screening and new drugs improved the therapeutic and preventive efficacy of esophageal cancer [23, 24]. Effective treatments and prevention of the Helicobacter pylori (HP) infection declined the death of stomach cancer [25, 26]. Endoscopic screening combined with occult blood testing were confirmed as an effective method to avert a significant amount of DALYs and YLLs [27], probably explained why the YLLs caused by colorectal cancer had a decreasing trend observed in the present article.

The findings that low-resource areas had higher YLLs than high-resource ones in the previous published article [28], demonstrated higher YLLs and YLDs caused by gastrointestinal cancers in low and middle SDI areas. Larger decreasing trends occurred in high SDI areas, probably attributed to the advances in treatment and detection of cancer, and medical sources were more available in these areas [29]. This could explain that EAPCs had negative association with HDI at the national level. At the national level, there existed high heterogeneity in trend of YLLs and YLDs, mainly involved with the factors, including local health sources, economic development, and so on. Pronounced decreasing trends of YLLs and YLDs caused by esophageal cancer were observed in the Central Asia countries, particularly Turkmenistan and Uzbekistan, probably attributed to the prevention and endoscopic management in early esophageal cancer [30], and the increasing proportion of patients with esophageal cancer diagnosed at an older age (61–70 years) [31]. Whereas pronounced increasing trends of YLLs and YLDs caused by esophageal cancer occurred in Taiwan (China), probably associated with the behaviors of chewing betel quid [32]. South Korea was one of the countries with the largest decreasing trends in YLLs of stomach cancer, in where high coverage of early endoscopic screening was reported [33], and Smoking rate in males fell from 71.7% in 1992 to 39.7% in 2016 [34]. The largest decreasing trend of YLLs caused by colorectal cancer was observed in Austria, in where early detection was more than the improvements of treatment [35]. YLDs caused by colorectal cancer had apparent increasing trends in China, which was mainly due to the pronounced decreasing mortality rate and prolonged survival of colorectal cancer [36]. Meanwhile, the highest increasing incident trend was observed in the youths (aged 15–49 years) [37], indicating that longer course with disability. Advances in cancer control had been shown to slow in most countries [38], and further policy actions and investments were needed to advancing the availability and quality of health care in low and middle SDI countries [39]. In the WHO Noncommunicable Diseases Plan, population-based screening, and early and timely treatments were recommended to be implemented in countries [40].

There are several limitations to this study. 1. The GBDs depend on the quality and quantity of data, and potential bias caused by misclassification and miscoding of the disease may weaken the robustness and accuracy of the GBD estimates. 2. The discrepancy of diagnostic standards existed across countries and over time, which also impaired the accuracy of estimation. 3. Data on YLLs and YLDs caused by three common gastrointestinal cancers was lack in some countries; thus the trends failed to be fully explained in those countries.

## Conclusions

YLLs and YLDs caused by esophageal cancer, stomach cancer, and colorectal cancer presented decreasing trends worldwide from 1990 to 2019, but existed pronounced heterogeneity in regions and countries. The disease burden of gastrointestinal cancers remains a substantial challenge globally, emphasizing the effective strategies of prevention and healthcare.

## Supplementary Information

**Additional file 1: Supplementary figure 1**. The distribution of ASR of YLLs and YLDs caused by esophageal cancer in SDI areas and geographic regions from1990 to 2019. (A) and (B) respectively presented the ASR of YLLs in SDI areas and geographic regions; (C) and (D) respectively presented the ASR of YLDs in SDI areas and geographic regions. YLLs: years of life lost; YLDs: years lived with disability; SDI: sociodemographic index. **Supplementary figure 2.** The distribution of ASR of YLLs and YLDs caused by stomach cancer in SDI areas and geographic regions from1990 to 2019. (A) and (B) respectively presented the ASR of YLLs in SDI areas and geographic regions; (C) and (D) respectively presented the ASR of YLDs in SDI areas and geographic regions. YLLs: years of life lost; YLDs: years lived with disability; SDI: sociodemographic index. **Supplementary figure 3.** The distribution of ASR of YLLs and YLDs caused by colorectal cancer in SDI areas and geographic regions from1990 to 2019. (A) and (B) respectively presented the ASR of YLLs in SDI areas and geographic regions; (C) and (D) respectively presented the ASR of YLDs in SDI areas and geographic regions. YLLs: years of life lost; YLDs: years lived with disability; SDI: sociodemographic index. **Supplementary figure 4.** The associations between ASRs of YLDs caused by gastrointestinal cancers and SDI in 2019 among regions. (A), (B) and (C) were ASR of YLDs caused by esophageal cancer, stomach cancer, and colorectal cancer, respectively. The association was calculated with Pearson correlation analysis. The symbols were the countries/territories in the corresponding regions. ASR, age-standardized rate; socio-demographic index Age-standardized rates; YLDs, years lived with disability. **Supplementary figure 5.** The distribution of percentage changes in number of YLLs caused by gastrointestinal cancers between 1990 and 2019 at the national level. (A), (B), and (C) respectively presented that of esophageal cancer, stomach cancer, and colorectal cancer. Countries/territories with an extreme value were annotated. YLLs: years of life lost. **Supplementary figure 6.** The distribution of percentage changes in number of YLDs caused by gastrointestinal cancers between 1990 and 2019 at the national level. (A), (B), and (C) respectively presented that of esophageal cancer, stomach cancer, and colorectal cancer. Countries/territories with an extreme value were annotated. YLDs: years lived with disability. **Supplementary figure 7.** The association between EAPCs of YLDs and ASR in 1990 at the national level. The EAPCs of YLDs caused by esophageal cancer (A), stomach cancer (B), and colorectal cancer (C) had negative associations with the corresponding ASR in 1990. The association was calculated with Pearson correlation analysis. The size of circle is increased with the numbers of YLDs in 1990. YLDs: years lived with disability; EAPC, estimated annual percentage change. **Supplementary table 1.** The percentage changes in number and EAPCs of YLLs and YLDs due to stomach cancer from 1990 to 2019 in global, sexes, SDI areas and geographic regions. **Supplementary table 2.** The percentage changes in number and EAPCs of YLLs and YLDs due to colorectal cancer from 1990 to 2019 in global, sexes, SDI areas and geographic regions. **Supplementary table 3.** The percentage changes in number and EAPCs of YLLs at national level and both sexes from 1990 to 2019. **Supplementary table 4.** The percentage change in number and EAPCs of YLDs at national level and both sexes from 1990 to 2019.

## Data Availability

All data in the study are available in the main manuscript and supplementary files. Public access to the database of GBD study is open, and data are available for download through the Global Health Data Exchange (GHDx) (http://ghdx.healthdata.org), which was created and supported by the Institute for Health Metrics and Evaluation (IHME) at the University of Washington.

## Abbreviations

GBD: Global Burden of Disease
YLLs: Years of life lost
YLDs: Years lived with disability
DALYs: Neonatal sepsis and other neonatal infections
EAPC: Estimated annual percentage change
GHDx: Global health data exchange
SDI: Socio-demographic index
ASR: Age-standardized rate
CI: Confidence interval
UI: Uncertainty interval
HDI: Human Development Index
