# Supplementary material for: Global and national trends in years of life lost and years lived with disability caused by three common gastrointestinal cancers from 1990 to 2019
Source: BMC Gastroenterol. 2022 Nov 28;22:493. doi: 10.1186/s12876-022-02567-5 (PMC9706987; doi:10.1186/s12876-022-02567-5)
Supplement: Supplementary file 1 — Additional file 1: Supplementary figure 1. The distribution of ASR of YLLs and YLDs caused by esophageal cancer in SDI areas and geographic regions from1990 to 2019. (A) and (B) respectively presented the ASR of YLLs in SDI areas and geographic regions; (C) and (D) respectively presented the ASR of YLDs in SDI areas and geographic regions. YLLs: years of life lost; YLDs: years lived with disability; SDI: sociodemographic index. Supplementary figure 2. The distribution of ASR of YLLs and YLDs caused by stomach cancer in SDI areas and geographic regions from1990 to 2019. (A) and (B) respectively presented the ASR of YLLs in SDI areas and geographic regions; (C) and (D) respectively presented the ASR of YLDs in SDI areas and geographic regions. YLLs: years of life lost; YLDs: years lived with disability; SDI: sociodemographic index. Supplementary figure 3. The distribution of ASR of YLLs and YLDs caused by colorectal cancer in SDI areas and geographic regions from1990 to 2019. (A) and (B) respectively presented the ASR of YLLs in SDI areas and geographic regions; (C) and (D) respectively presented the ASR of YLDs in SDI areas and geographic regions. YLLs: years of life lost; YLDs: years lived with disability; SDI: sociodemographic index. Supplementary figure 4. The associations between ASRs of YLDs caused by gastrointestinal cancers and SDI in 2019 among regions. (A), (B) and (C) were ASR of YLDs caused by esophageal cancer, stomach cancer, and colorectal cancer, respectively. The association was calculated with Pearson correlation analysis. The symbols were the countries/territories in the corresponding regions. ASR, age-standardized rate; socio-demographic index Age-standardized rates; YLDs, years lived with disability. Supplementary figure 5. The distribution of percentage changes in number of YLLs caused by gastrointestinal cancers between 1990 and 2019 at the national level. (A), (B), and (C) respectively presented that of esophageal cancer, stomach cancer, and co [file 12876_2022_2567_MOESM1_ESM.docx]

**Supplementary figure 1.** The distribution of ASR of YLLs and YLDs caused by esophageal cancer in SDI areas and geographic regions from1990 to 2019. (A) and (B) respectively presented the ASR of YLLs in SDI areas and geographic regions; (C) and (D) respectively presented the ASR of YLDs in SDI areas and geographic regions. YLLs: years of life lost; YLDs: years lived with disability; SDI: sociodemographic index.

**
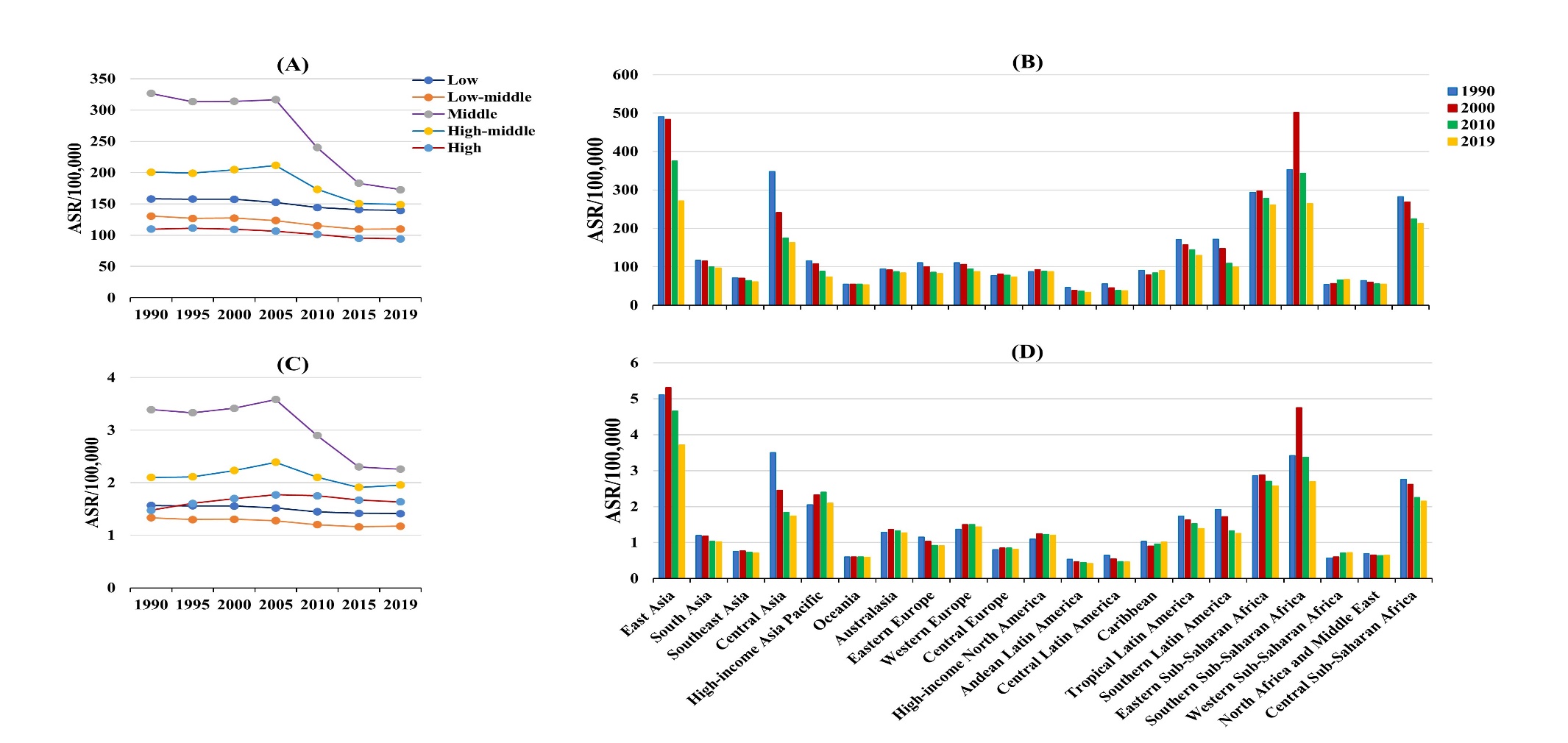
**

**Supplementary figure 2.** The distribution of ASR of YLLs and YLDs caused by stomach cancer in SDI areas and geographic regions from1990 to 2019. (A) and (B) respectively presented the ASR of YLLs in SDI areas and geographic regions; (C) and (D) respectively presented the ASR of YLDs in SDI areas and geographic regions. YLLs: years of life lost; YLDs: years lived with disability; SDI: sociodemographic index.

**
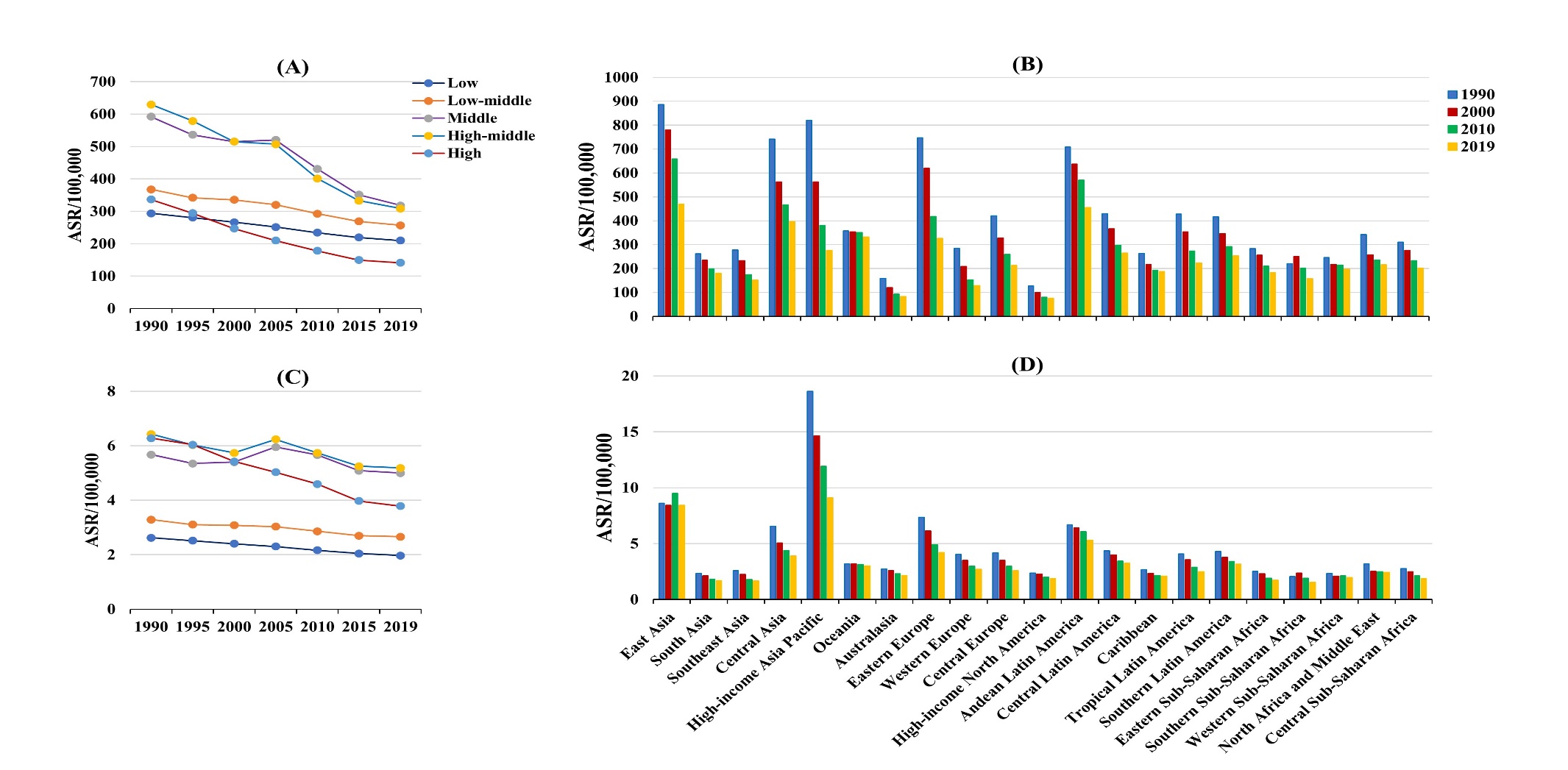
**

**Supplementary figure 3.** The distribution of ASR of YLLs and YLDs caused by colorectal cancer in SDI areas and geographic regions from1990 to 2019. (A) and (B) respectively presented the ASR of YLLs in SDI areas and geographic regions; (C) and (D) respectively presented the ASR of YLDs in SDI areas and geographic regions. YLLs: years of life lost; YLDs: years lived with disability; SDI: sociodemographic index.

**
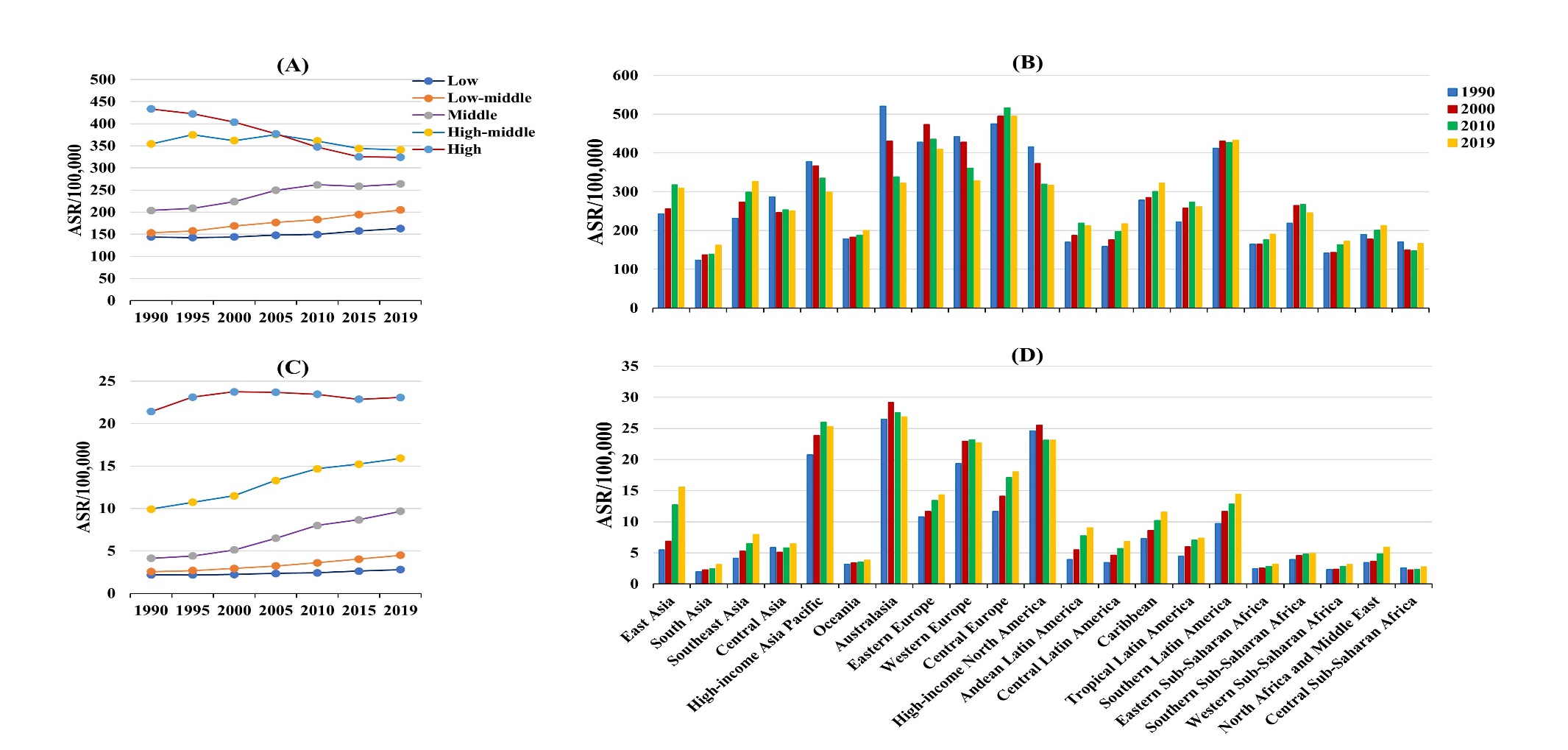
**

**Supplementary figure 4**. The associations between ASRs of YLDs caused by gastrointestinal cancers and SDI in 2019 among regions. (A), (B) and (C) were ASR of YLDs caused by esophageal cancer, stomach cancer, and colorectal cancer, respectively. The association was calculated with Pearson correlation analysis. The symbols were the countries/territories in the corresponding regions. ASR, age-standardized rate; socio-demographic index Age-standardized rates; YLDs, years lived with disability.

**
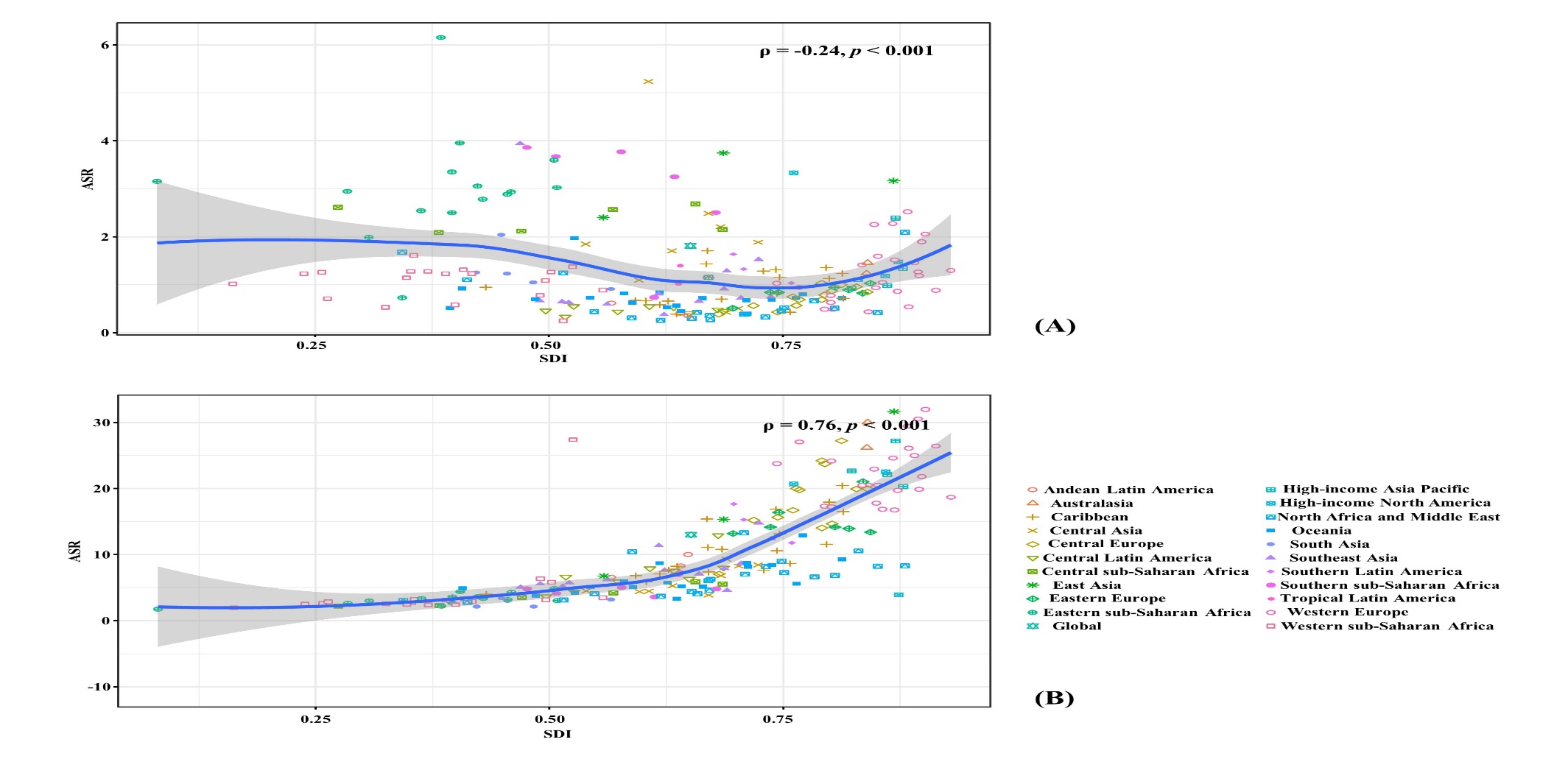
**

**Supplementary figure 5.** The distribution of percentage changes in number of YLLs caused by gastrointestinal cancers between 1990 and 2019 at the national level. (A), (B), and (C) respectively presented that of esophageal cancer, stomach cancer, and colorectal cancer. Countries/territories with an extreme value were annotated. YLLs: years of life lost.

**
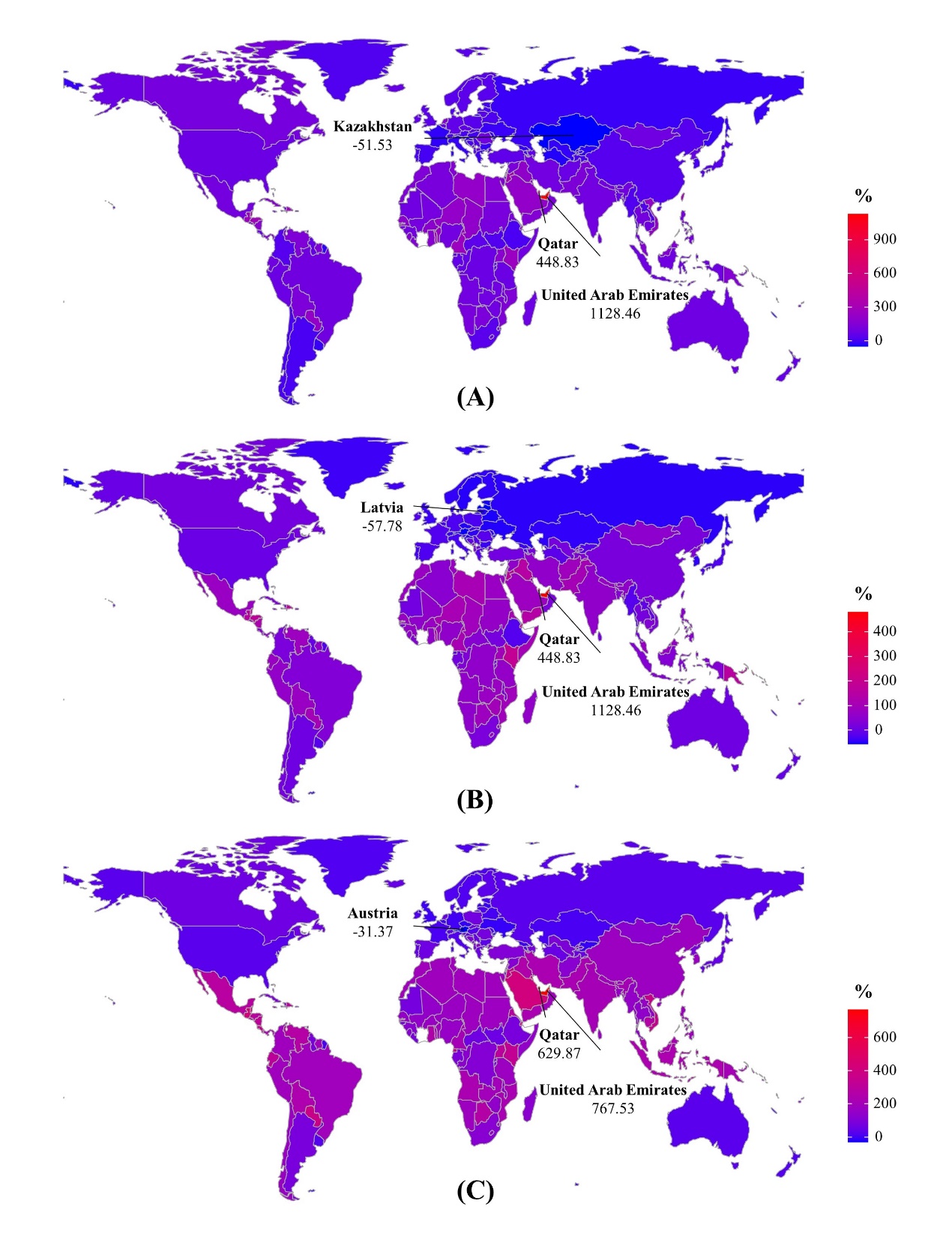
**

**Supplementary figure 6.** The distribution of percentage changes in number of YLDs caused by gastrointestinal cancers between 1990 and 2019 at the national level. (A), (B), and (C) respectively presented that of esophageal cancer, stomach cancer, and colorectal cancer. Countries/territories with an extreme value were annotated. YLDs: years lived with disability.

**
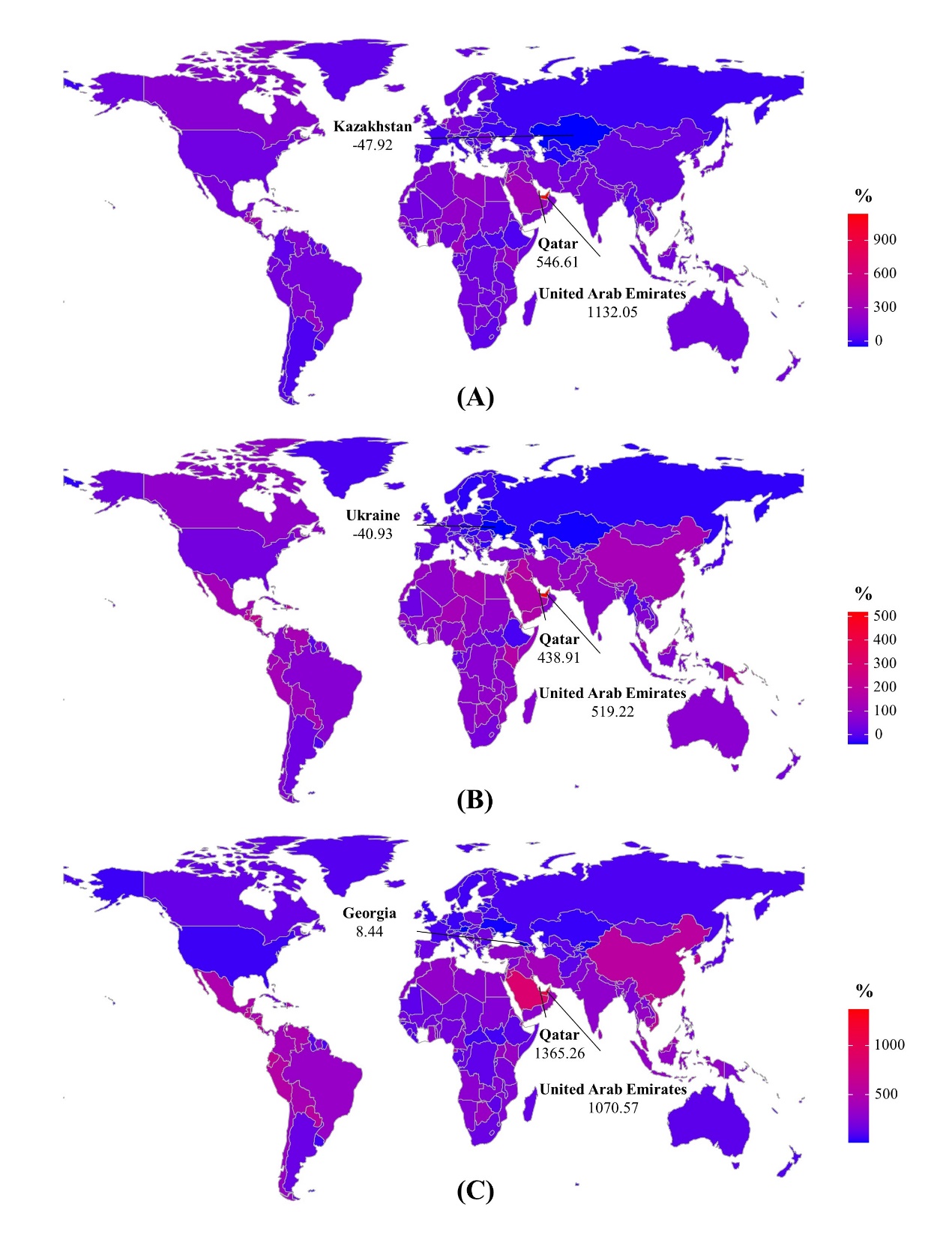
**

**Supplementary figure 7.** The association between EAPCs of YLDs and ASR in 1990 at the national level. The EAPCs of YLDs caused by esophageal cancer (A), stomach cancer (B), and colorectal cancer (C) had negative associations with the corresponding ASR in 1990. The association was calculated with Pearson correlation analysis. The size of circle is increased with the numbers of YLDs in 1990. YLDs: years lived with disability; EAPC, estimated annual percentage change.

**
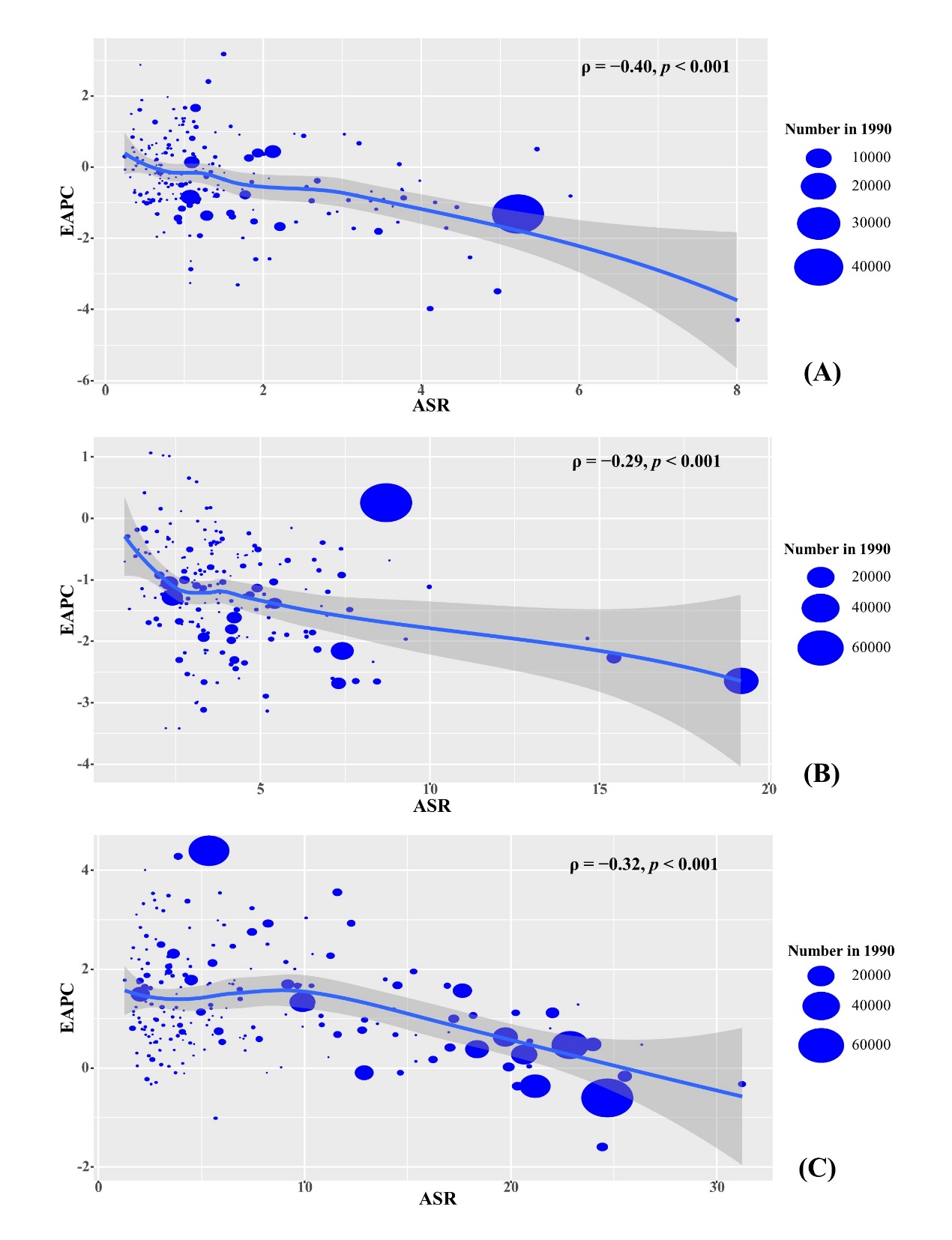
**

**Supplementary table 1**. The percentage changes in number and EAPCs of YLLs and YLDs due to stomach cancer from 1990 to 2019 in global, sexes, SDI areas and geographic regions

| **Characteristics** | **YLLs** | | | | **YLDs** | | | |
| --- | --- | --- | --- | --- | --- | --- | --- | --- |
|  | 2019 | | 1990−2019 | | 2019 | | 1990−2019 | |
|  | Number  ×10^3^ (95% UI) | ASR/100,000  (95% UI) | Percentage change in  number (%) | EAPCs  (95%CI) | Number  ×10^3^ (95% UI) | ASR/100,000  (95% UI) | Percentage change in  number (%) | EAPCs  (95%CI) |
| **Overall** | 21872.43  (19972.71-23712.52) | 264.15  (241.47-286.03) | 8.06 | −2.13  (−2.29-−1.96) | 348.55  (252.33-457.64) | 4.25  (3.08-5.57) | 57.47 | −0.85  (−0.97-−0.73) |
| **Sex** |  |  |  |  |  |  |  |  |
| Male | 14283.66  (12662.99-15951.75) | 362.68  (322.15-403.44) | 12.91 | −1.95  (−2.13-−1.76) | 237.51  (169.74-312.8) | 6.17  (4.43-8.11) | 70.77 | −0.56  (−0.7-−0.42) |
| Female | 7588.77  (6818.76-8387.97) | 175.7  (157.9-194.11) | −0.03 | −2.48  (−2.6-−2.35) | 111.04  (79.87-145.79) | 2.55  (1.83-3.35) | 34.98 | −1.48  (−1.57-−1.38) |
| **SDI** |  |  |  |  |  |  |  |  |
| Low | 1222.71  (1083.91-1377.37) | 210.02  (187.28-234.84) | 56.15 | −1.2  (−1.23-−1.17) | 10.54  (7.46-13.97) | 1.97  (1.41-2.59) | 63.31 | −1.01  (−1.04-−0.98) |
| Low-middle | 3736.47  (3407.39-4089.3) | 256.98  (235.16-280.48) | 50.56 | −1.24  (−1.32-−1.15) | 37.04  (26.09-48.27) | 2.66  (1.89-3.46) | 79.13 | −0.74  (−0.81-−0.68) |
| Middle | 8147.6  (7151.11-9182.61) | 318.39  (280.47-357.79) | 22.9 | −2.03  (−2.3-−1.76) | 125.67  (89.94-167.59) | 5  (3.59-6.65) | 108.71 | −0.25  (−0.45-−0.05) |
| High-middle | 6247.74  (5556.19-6913.6) | 308.87  (274.88-341.87) | −10.01 | −2.54  (−2.75-−2.32) | 105.01  (75.75-137.81) | 5.18  (3.74-6.81) | 50.99 | −0.64  (−0.8-−0.48) |
| High | 2509.77  (2326.77-2628.36) | 141.52  (133.16-147.74) | −26.16 | −3.16  (−3.22-−3.10) | 70.19  (50.92-90.85) | 3.79  (2.76-4.91) | 9.03 | −1.87  (−1.95-−1.80) |
| **Regions** |  |  |  |  |  |  |  |  |
| East Asia | 9925.74  (8292.1-11718.76) | 469.5  (393.81-551.74) | 18.32 | −2.00  (−2.39-−1.61) | 177.04  (124.5-238.37) | 8.43  (5.96-11.38) | 127.3 | 0.24  (0.06-0.54) |
| South Asia | 2749.98  (2392.19-3140.36) | 180.36  (157.03-205.74) | 58.8 | −1.36  (−1.43-−1.28) | 24.53  (17.25-32.67) | 1.69  (1.19-2.24) | 74.52 | −1.18  (−1.26-−1.11) |
| Southeast Asia | 977.16  (866.74-1093.82) | 151.86  (135.18-169.84) | 23.2 | −2.30  (−2.39-−2.22) | 10.17  (7.17-13.5) | 1.66  (1.17-2.2) | 48.55 | −1.73  (−1.82-−1.65) |
| Central Asia | 322  (290.99-356.93) | 396.97  (360.5-438.31) | −13.24 | −2.21  (−2.33-−2.1) | 2.97  (2.08-3.97) | 3.89  (2.75-5.16) | −6.89 | −1.81  (−1.93-−1.68) |
| High-income Asia Pacific | 1122.34  (1010.28-1191.69) | 275.6  (255.53-290.03) | −32.98 | −3.97  (−4.04-−3.89) | 39.01  (27.74-51.48) | 9.09  (6.51-12.07) | 3.04 | −2.58  (−2.67-−2.48) |
| Oceania | 28.44  (21.41-36.41) | 331.99  (253-418.71) | 123 | −0.21  (−0.26-−0.17) | 0.23  (0.15-0.34) | 3.01  (1.99-4.29) | 125.06 | −0.2  (−0.24-−0.16) |
| Australasia | 37.87  (35.07-40.39) | 82.34  (76.78-87.64) | 2.62 | −2.37  (−2.49-−2.25) | 1.04  (0.7-1.44) | 2.16  (1.45-2.99) | 61.19 | −0.94  (−1.02-−0.87) |
| Eastern Europe | 1067.26  (959.82-1188.29) | 326.19  (293.08-362.58) | −49.24 | −3.45  (−3.70-−3.19) | 13.97  (10.07-18.09) | 4.21  (3.03-5.46) | −32.43 | −2.26  (−2.45-−2.07) |
| Western Europe | 1073.88  (1005.12-1123.72) | 129.27  (122.76-134.94) | −32.2 | −2.83  (−2.92-−2.75) | 23.6  (16.81-30.88) | 2.71  (1.92-3.58) | 1.54 | −1.49  (−1.54-−1.44) |
| Central Europe | 422.96  (370.37-476.36) | 212.48  (185.31-240.07) | −31.92 | −2.43  (−2.49-−2.37) | 5.34  (3.8-7.01) | 2.58  (1.83-3.39) | −13.13 | −1.7  (−1.75-−1.65) |
| High-income North America | 434.86  (416.07-449.23) | 75.49  (72.78-77.87) | 0.41 | −1.93  (−2.05-−1.81) | 11.33  (7.97-14.79) | 1.89  (1.32-2.48) | 36.76 | −0.96  (−1.04-−0.89) |
| Andean Latin America | 260.08  (210.23-317.16) | 455.88  (369.52-555.54) | 67.69 | −1.55  (−1.67-−1.44) | 2.95  (1.98-4.12) | 5.3  (3.55-7.4) | 113.24 | −0.82  (−0.91-−0.73) |
| Central Latin America | 640.18  (545.36-754.11) | 265.13  (226.03-311.82) | 66.4 | −1.92  (−2-−1.83) | 7.69  (5.35-10.29) | 3.24  (2.25-4.33) | 110 | −1.28  (−1.37-−1.19) |
| Caribbean | 96.61  (81.98-111.94) | 187.45  (158.81-217.11) | 37.1 | −1.07  (−1.2-−0.95) | 1.09  (0.74-1.48) | 2.11  (1.44-2.86) | 54.92 | −0.73  (−0.82-−0.63) |
| Tropical Latin America | 550.68  (524.15-575.27) | 223.18  (212.14-233.22) | 31.97 | −2.29  (−2.36-−2.22) | 6.04  (4.34-7.76) | 2.49  (1.79-3.19) | 60.05 | −1.78  (−1.86-−1.71) |
| Southern Latin America | 206.66  (195.77-217.36) | 253.35  (240.52-266.21) | 6.57 | −1.74  (−1.79-−1.68) | 2.61  (1.72-3.68) | 3.16  (2.08-4.45) | 32.46 | −1.07  (−1.13-−1.01) |
| Eastern Sub-Saharan Africa | 342.87  (295.5-396.6) | 182.68  (159.4-208.79) | 39.83 | −1.69  (−1.75-−1.62) | 2.91  (2.04-3.91) | 1.71  (1.21-2.29) | 45.3 | −1.47  (−1.54-−1.41) |
| Southern Sub-Saharan Africa | 94.84  (85.73-105.61) | 156.86  (142.87-173.23) | 40.84 | −1.27  (−1.59-−0.94) | 0.88  (0.63-1.17) | 1.55  (1.1-2.04) | 50.2 | −1.11  (−1.42-−0.81) |
| Western Sub-Saharan Africa | 392.67  (334.04-459.22) | 197.74  (169.94-227.54) | 71.6 | −0.58  (−0.65-−0.51) | 3.57  (2.45-4.83) | 1.97  (1.38-2.64) | 77.03 | −0.38  (−0.45-−0.3) |
| North Africa  and Middle East | 1000.92  (895.99-1120.11) | 215.7  (194.65-239.85) | 54.46 | −1.51  (−1.7-−1.31) | 10.53  (7.3-13.91) | 2.43  (1.71-3.19) | 86.34 | −0.85  (−1.05-−0.64) |
| Central Sub-Saharan Africa | 124.45  (97.01-156.54) | 202.23  (161.56-251.19) | 54.16 | −1.54  (−1.6-−1.49) | 1.05  (0.7-1.51) | 1.89  (1.27-2.67) | 59.44 | −1.36  (−1.41-−1.32) |

YLLs: years of life lost; YLDs: years lived with disability; EAPC: estimated annual percentage change; ASR, age-standardized rate; CI, confidence interval; UI: uncertainty interval; SDI: socio-demographic index.

**Supplementary table 2**. The percentage changes in number and EAPCs of YLLs and YLDs due to colorectal cancer from 1990 to 2019 in global, sexes, SDI areas and geographic regions

| **Characteristics** | **YLLs** | | | | **YLDs** | | | |
| --- | --- | --- | --- | --- | --- | --- | --- | --- |
|  | 2019 | | 1990−2019 | | 2019 | | 1990−2019 | |
|  | Number  ×10^3^ (95% UI) | ASR/100,000  (95% UI) | Percentage change in  number (%) | EAPCs  (95%CI) | Number  ×10^3^ (95% UI) | ASR/100,000  (95% UI) | Percentage change in  number (%) | EAPCs  (95%CI) |
| **Overall** | 23218.75  (21662.64-24591.16) | 282.51  (263.5-299.24) | 93.28 | −0.25  (−0.30-−0.19) | 1065.34  (779.13-1378.75) | 13.03  (9.54-16.82) | 169.70 | 0.83  (0.77-0.89) |
| **Sex** |  |  |  |  |  |  |  |  |
| Male | 13338.45  (12294.7-14444.06) | 343.77  (317.1-371.07) | 112.73 | 0.09  (0.02-0.16) | 621.14  (454.11-806.45) | 16.28  (11.96-21.13) | 205.24 | 1.21  (1.14-1.28) |
| Female | 9880.3  (9089.04-10712.09) | 227.74  (209.51-246.84) | 72.04 | −0.69  (−0.74-−0.64) | 444.19  (324.59-577.67) | 10.19  (7.44-13.26) | 131.94 | 0.32  (0.26-0.38) |
| **SDI** |  |  |  |  |  |  |  |  |
| Low | 927.46  (823.29-1042.42) | 163.3  (145.77-182.73) | 146.85 | −0.25  (−0.36-−0.13) | 14.96  (10.93-19.89) | 2.8  (2.05-3.71) | 180.27 | 0.88  (0.79-0.97) |
| Low-middle | 2935.64  (2648.85-3248.81) | 205.02  (185.04-226.74) | 188.39 | 0.20  (0.15−0.25) | 62.68  (45.98-81.93) | 4.49  (3.31-5.86) | 289.64 | 1.97  (1.9-2.04) |
| Middle | 6745.06  (6074.97-7415.09) | 263.93  (237.41-289.41) | 190.58 | 0.19  (0.13−0.26) | 245.37  (177.07-325.39) | 9.68  (7.03-12.78) | 449.6 | 3.35  (3.18-3.53) |
| High-middle | 6852.95  (6317.18-7358.11) | 340.74  (314.43-365.75) | 77.8 | −0.54  (−0.63-−0.45) | 321.91  (234.81-420.16) | 15.91  (11.6-20.77) | 203.26 | 1.78  (1.67-1.89) |
| High | 5744.74  (5389.74-5981.5) | 324.23  (307.7-336.17) | 29.45 | −1.25  (−1.31-−1.19) | 419.92  (306.11-542.65) | 23.08  (16.79-29.88) | 88.64 | 0.09  (−0.02-0.19) |
| **Regions** |  |  |  |  |  |  |  |  |
| East Asia | 6388.31  (5470.03-7429.16) | 309.63  (265.77-358.33) | 173.57 | 1.14  (0.91-1.36) | 324.56  (229.48-436.03) | 15.55  (11.03-20.92) | 544.85 | 4.32  (4.01-4.62) |
| South Asia | 2374.78  (2033-2741.45) | 161.95  (138.84-186.97) | 206.36 | 0.79  (0.67-0.91) | 44.32  (31.69-59.62) | 3.12  (2.23-4.17) | 283.89 | 1.47  (1.35-1.59) |
| Southeast Asia | 2092.38  (1739.42-2423.47) | 326.03  (269.4-377.14) | 216.27 | 1.09  (1.03-1.16) | 50.05  (34.88-66.18) | 7.94  (5.54-10.47) | 343.03 | 2.19  (2.16-2.23) |
| Central Asia | 194.99  (177.75-214.3) | 250.38  (228.95-274.23) | 34.53 | −0.39  (−0.55-−0.24) | 4.85  (3.52-6.37) | 6.43  (4.69-8.37) | 68.69 | 0.53  (0.3-0.75) |
| High-income Asia Pacific | 1220.62  (1093.2-1293.9) | 298.59  (276.31-312.96) | 59.63 | −0.98  (−1.06-−0.9) | 107.2  (77.1-142.85) | 25.29  (18.12-33.72) | 156.43 | 0.57  (0.45-0.69) |
| Oceania | 16.03  (12.68-20.18) | 199.76  (160.43-246.91) | 162.65 | 0.35  (0.29-0.42) | 0.29  (0.19-0.4) | 3.81  (2.58-5.18) | 183.44 | 0.57  (0.5-0.63) |
| Australasia | 150.34  (139.7-159.02) | 321.76  (301.39-339.57) | 25.31 | −2.00  (−2.16-−1.84) | 12.9  (8.96-17.59) | 26.84  (18.53-36.75) | 108.93 | −0.20  (−0.33-−0.07) |
| Eastern Europe | 1370.8  (1236.86-1519.04) | 409.36  (369.75-453.92) | 14.13 | −0.61  (−0.84-−0.38) | 48.31  (34.73-63.45) | 14.35  (10.31-18.91) | 59.7 | 0.95  (0.82-1.08) |
| Western Europe | 2810.59  (2625.01-2925.79) | 328.55  (310.76-340.58) | 13.23 | −1.26  (−1.39-−1.12) | 197.64  (141.79-260.07) | 22.69  (16.23-30.07) | 78.21 | 0.4  (0.21-0.59) |
| Central Europe | 1014.7  (891.28-1140.33) | 494.53  (433.82-558.13) | 45.09 | 0.13  (0.03-0.23) | 37.44  (27.22-49.21) | 18.03  (13.04-23.7) | 118.3 | 1.62  (1.5-1.74) |
| High-income North America | 1848.88  (1771.24-1902.95) | 316.77  (304.77-325.21) | 30.01 | −1.11  (−1.21-−1.01) | 138.23  (99.99-181.1) | 23.09  (16.66-30.44) | 60.13 | −0.48  (−0.58-−0.38) |
| Andean Latin America | 120.51  (97.49-145.75) | 211.74  (170.64-255.55) | 223.86 | 0.97  (0.82-1.12) | 5.07  (3.5-7) | 9.03  (6.21-12.45) | 514.51 | 3.18  (2.99-3.38) |
| Central Latin America | 523.42  (451.47-607.84) | 216.87  (187.34-251.56) | 260.58 | 1.08  (1.04-1.12) | 16.22  (11.43-21.8) | 6.81  (4.79-9.12) | 440.84 | 2.27  (2.17-2.36) |
| Caribbean | 166.06  (142.07-192.8) | 321.77  (275.06-373.81) | 124.34 | 0.57  (0.5-0.63) | 5.96  (4.28-7.86) | 11.53  (8.29-15.21) | 211.74 | 1.7  (1.63-1.77) |
| Tropical Latin America | 642.14  (608.84-668.75) | 260.92  (246.96-271.81) | 194.65 | 0.65  (0.48-0.83) | 17.99  (13.28-23.2) | 7.37  (5.46-9.53) | 332.3 | 1.82  (1.59-2.04) |
| Southern Latin America | 354.54  (336.92-371.89) | 433.14  (411.8-453.81) | 85.79 | 0.13  (0.06-0.21) | 11.9  (8.18-16.53) | 14.41  (9.89-20.07) | 165.8 | 1.32  (1.23-1.41) |
| Eastern Sub-Saharan Africa | 350.95  (297.91-419.17) | 190.68  (163.16-225.85) | 153.9 | 0.54  (0.46-0.61) | 5.48  (3.81-7.68) | 3.19  (2.25-4.38) | 182.51 | 0.91  (0.82-1) |
| Southern Sub-Saharan Africa | 145.01  (129.78-162.49) | 245.56  (220.44-274.39) | 124.54 | 0.49  (0.27-0.7) | 2.77  (2-3.72) | 4.87  (3.51-6.51) | 152.36 | 0.83  (0.72-0.95) |
| Western Sub-Saharan Africa | 347.43  (290.07-414.4) | 173.01  (146.45-203.13) | 165.38 | 0.88  (0.79-0.97) | 5.81  (4.09-7.74) | 3.1  (2.19-4.08) | 189.38 | 1.23  (1.12-1.35) |
| North Africa  and Middle East | 986.8  (873.74-1117.44) | 212.75  (188.31-239.48) | 174.94 | 0.52  (0.34-0.7) | 26.83  (19.53-35.8) | 5.93  (4.34-7.84) | 330.44 | 2.07  (1.87-2.27) |
| Central Sub-Saharan Africa | 99.47  (74.53-129.54) | 166.52  (126.81-216.06) | 131.36 | −0.15  (−0.39-0.10) | 1.52  (1-2.17) | 2.75  (1.82-3.97) | 154.28 | 0.18  (−0.07-0.44) |

YLLs: years of life lost; YLDs: years lived with disability; EAPC: estimated annual percentage change; ASR, age-standardized rate; CI, confidence interval; UI: uncertainty interval; SDI: socio-demographic index.

**Supplementary table 3**. The percentage changes in number and EAPCs of YLLs at national level and both sexes from 1990 to 2019

| **Characteristics** | **Esophageal cancer** | | **Stomach cancer** | | **Colorectal cancer** | | |
| --- | --- | --- | --- | --- | --- | --- | --- |
|  | Percentage change in  number (%) | EAPC  (95%CI) | Percentage change in  number (%) | EAPC  (95%CI) | Percentage change in  number (%) | EAPC  (95%CI) | |
| Afghanistan | 70.89 | −0.55(−0.69-−0.4) | 86.17 | −0.48(−0.71-−0.25) | 118.56 | 0.31(0.12-0.5) |  |
| Albania | 18.91 | −1.23(−1.55-−0.9) | 15.86 | −1.54(−1.75-−1.33) | 81.14 | 0.44(0.16-0.72) |  |
| Algeria | 115.56 | −0.97(−1.11-−0.83) | 43.51 | −2.24(−2.38-−2.1) | 182.9 | 0.19(0.15-0.23) |  |
| American Samoa | 126.93 | 0.5(0.33-0.67) | 46.61 | −0.85(−1.01-−0.68) | 98.06 | 0.13(0-0.26) |  |
| Andorra | 102.79 | −0.76(−0.82-−0.7) | 62.06 | −1.53(−1.66-−1.41) | 96.81 | −0.91(−0.96-−0.87) |  |
| Angola | 94.89 | −1.57(−1.69-−1.44) | 62.45 | −2.04(−2.11-−1.96) | 223.97 | 0.56(0.38-0.74) |  |
| Antigua and Barbuda | 80.36 | −0.52(−0.77-−0.26) | 20.05 | −1.79(−1.99-−1.6) | 139.2 | 0.66(0.53-0.79) |  |
| Argentina | 4.68 | −1.82(−1.97-−1.68) | 5.08 | −1.53(−1.6-−1.46) | 81.34 | 0.27(0.15-0.39) |  |
| Armenia | −7.27 | −1.5(−1.81-−1.2) | −30.43 | −2.57(−2.75-−2.38) | 23.32 | −0.57(−0.7-−0.45) |  |
| Australia | 83.12 | −0.45(−0.52-−0.37) | 5.06 | −2.31(−2.43-−2.18) | 26.5 | −2.02(−2.2-−1.85) |  |
| Austria | 6.17 | −1.36(−1.47-−1.25) | −56.77 | −4.57(−4.82-−4.32) | −31.37 | −3.17(−3.33-−3.01) |  |
| Azerbaijan | 52.5 | −0.87(−1.06-−0.67) | 14.14 | −1.74(−1.95-−1.54) | 80 | 0.3(−0.2-0.81) |  |
| Bahamas | 96.53 | −0.72(−0.92-−0.51) | 51.79 | −1.73(−1.88-−1.58) | 151.16 | 0.38(0.29-0.47) |  |
| Bahrain | 148.27 | −3.83(−4.16-−3.49) | 98.13 | −3.62(−3.87-−3.36) | 331.01 | −0.98(−1.23-−0.73) |  |
| Bangladesh | 62.92 | −1.59(−1.72-−1.46) | 38.19 | −1.96(−2.13-−1.8) | 155.91 | −0.09(−0.19-0.02) |  |
| Barbados | 51.33 | −0.83(−0.96-−0.71) | 3.55 | −2.01(−2.16-−1.87) | 124.06 | 0.91(0.74-1.08) |  |
| Belarus | 14.51 | −0.73(−0.92-−0.53) | −53.65 | −3.94(−4.18-−3.7) | 5.63 | −1.3(−1.6-−1) |  |
| Belgium | 57.36 | 0.31(0.09-0.52) | −37.38 | −2.72(−2.89-−2.54) | −3.93 | −1.58(−1.65-−1.51) |  |
| Belize | 306.33 | 0.65(0.29-1.01) | 164.46 | −0.96(−1.44-−0.47) | 394.7 | 1.39(0.94-1.84) |  |
| Benin | 235.45 | 1.39(1.21-1.57) | 83.44 | −0.77(−0.9-−0.63) | 182.61 | 0.48(0.45-0.52) |  |
| Bermuda | 9.42 | −1.73(−2.03-−1.44) | −27.31 | −3.39(−3.61-−3.18) | 29.79 | −1.47(−1.61-−1.33) |  |
| Bhutan | 67.45 | −0.78(−0.93-−0.64) | 53.67 | −0.93(−0.99-−0.86) | 184.28 | 1.02(0.99-1.06) |  |
| Bolivia  (Plurinational State of) | 126.44 | −0.69(−0.8-−0.58) | 76.44 | −1.51(−1.61-−1.42) | 238.8 | 0.79(0.75-0.84) |  |
| Bosnia and Herzegovina | 7.09 | −1.04(−1.18-−0.91) | −9.15 | −1.5(−1.7-−1.3) | 95.39 | 1.6(1.35-1.85) |  |
| Botswana | 123.97 | −1.12(−1.63-−0.6) | 83.94 | −1.6(−2.06-−1.14) | 262.35 | 0.97(0.61-1.33) |  |
| Brazil | 91.8 | −0.95(−1.01-−0.89) | 31.04 | −2.31(−2.37-−2.24) | 192.03 | 0.62(0.44-0.79) |  |
| Brunei Darussalam | 79.36 | −2.09(−2.35-−1.83) | 9.82 | −3.28(−3.55-−3.01) | 174.79 | 0.43(0.13-0.73) |  |
| Bulgaria | −20.03 | −0.12(−0.78-0.55) | −41.59 | −1.18(−1.57-−0.78) | 30.9 | 1.84(1.38-2.31) |  |
| Burkina Faso | 180.88 | 1.27(1.09-1.46) | 73.5 | −0.56(−0.82-−0.3) | 162.98 | 0.65(0.48-0.83) |  |
| Burundi | 36.42 | −1.93(−2.11-−1.75) | 30.92 | −1.96(−2.1-−1.82) | 75.76 | −0.73(−0.85-−0.62) |  |
| Cabo Verde | 237.46 | 1.02(0.68-1.36) | 33.16 | −1.77(−2.22-−1.32) | 308.39 | 2.12(1.86-2.39) |  |
| Cambodia | 91.22 | −1.13(−1.28-−0.99) | 33.41 | −2.3(−2.42-−2.19) | 262.7 | 1.33(1.29-1.38) |  |
| Cameroon | 257.26 | 1.28(1.1-1.46) | 112.13 | −0.61(−0.72-−0.49) | 209.04 | 0.46(0.41-0.5) |  |
| Canada | 106.76 | 0.02(−0.06-0.1) | 11.7 | −2.12(−2.25-−1.99) | 59.42 | −0.9(−0.96-−0.84) |  |
| Central African Republic | 43.92 | −1.09(−1.19-−0.99) | 39.32 | −1.2(−1.25-−1.14) | 64.47 | −0.47(−0.53-−0.4) |  |
| Chad | 203.32 | 1.77(1.58-1.97) | 92.32 | 0.07(−0.03-0.17) | 178.71 | 1.16(1.09-1.23) |  |
| Chile | 9.22 | −3.37(−3.6-−3.14) | 14.26 | −2.47(−2.55-−2.39) | 162.8 | 0.67(0.55-0.8) |  |
| China | 27.74 | −2.28(−2.77-−1.79) | 18.09 | −2.01(−2.41-−1.62) | 173.6 | 1.14(0.9-1.38) |  |
| Colombia | 26.72 | −3.33(−3.55-−3.12) | 22.62 | −3.02(−3.17-−2.88) | 188.63 | 0.14(0.04-0.24) |  |
| Comoros | 69.82 | −1.25(−1.46-−1.04) | 52.91 | −1.5(−1.7-−1.31) | 136.6 | 0.1(−0.06-0.25) |  |
| Congo | 60.85 | −1.83(−2.02-−1.63) | 29.42 | −2.51(−2.68-−2.35) | 126.73 | −0.41(−0.61-−0.22) |  |
| Cook Islands | 45.79 | −1.03(−1.13-−0.94) | 1.86 | −1.78(−1.99-−1.56) | 61.6 | −0.48(−0.72-−0.23) |  |
| Costa Rica | 71.43 | −2.25(−2.51-−1.99) | 42.59 | −2.81(−3.09-−2.52) | 344.4 | 1.77(1.63-1.91) |  |
| Croatia | −22.81 | −1.52(−1.66-−1.39) | −49.5 | −3.18(−3.31-−3.04) | 39.39 | 0.41(0.23-0.59) |  |
| Cuba | 165.53 | 1.82(1.62-2.01) | 29.78 | −0.95(−1.05-−0.85) | 94.39 | 0.24(0.08-0.39) |  |
| Cyprus | 121.95 | −0.02(−0.3-0.25) | 72.78 | −0.85(−1.07-−0.63) | 141.63 | 0.24(0.04-0.44) |  |
| Czechia | 48.28 | 0.12(−0.02-0.26) | −51.08 | −3.76(−3.85-−3.67) | −11.87 | −1.98(−2.16-−1.8) |  |
| Cote d'Ivoire | 224.42 | 0.92(0.79-1.05) | 81.77 | −1.1(−1.18-−1.03) | 158.85 | −0.25(−0.38-−0.12) |  |
| Democratic People's Republic of Korea | 61.78 | −0.53(−0.61-−0.46) | 29.42 | −1.2(−1.32-−1.09) | 85.74 | 0.15(0.1-0.21) |  |
| Democratic Republic of  the Congo | 76.52 | −1.05(−1.13-−0.96) | 58.15 | −1.31(−1.36-−1.25) | 108.46 | −0.48(−0.79-−0.16) |  |
| Denmark | 19.97 | −0.77(−0.89-−0.65) | −39.74 | −2.71(−2.94-−2.49) | 15.22 | −1.18(−1.41-−0.95) |  |
| Djibouti | 255.36 | −0.76(−0.9-−0.62) | 217.24 | −0.98(−1.1-−0.85) | 402.36 | 0.73(0.67-0.78) |  |
| Dominica | 30.2 | 0.05(−0.13-0.22) | −11.58 | −1.34(−1.48-−1.2) | 52.41 | 0.77(0.71-0.82) |  |
| Dominican Republic | 254.55 | 1.88(1.69-2.07) | 165.1 | 0.83(0.67-0.99) | 297.1 | 2.39(2.22-2.56) |  |
| Ecuador | 88.66 | −1.21(−1.43-−0.99) | 74.8 | −1.47(−1.7-−1.24) | 306.68 | 1.91(1.62-2.19) |  |
| Egypt | 155.55 | 0.68(0.58-0.78) | 76.29 | −0.45(−0.55-−0.36) | 182.62 | 1.01(0.93-1.08) |  |
| El Salvador | 105 | −0.2(−0.55-0.16) | 69.1 | −0.96(−1.31-−0.61) | 238.94 | 1.91(1.57-2.25) |  |
| Equatorial Guinea | 51.7 | −1.71(−2.01-−1.42) | −6.09 | −3.62(−3.98-−3.26) | 378.42 | 3.04(2.78-3.30) |  |
| Eritrea | 119.24 | −0.82(−1.03-−0.61) | 109.34 | −0.87(−0.99-−0.74) | 279.77 | 1.03(0.76-1.30) |  |
| Estonia | −5.37 | −0.6(−0.84-−0.35) | −55.1 | −3.55(−3.73-−3.36) | 2.26 | −0.8(−1-−0.6) |  |
| Eswatini | 90.88 | −0.29(−0.96-0.38) | 66.54 | −0.33(−0.81-0.16) | 168.99 | 1.19(0.75-1.63) |  |
| Ethiopia | 7.88 | −2.33(−2.46-−2.19) | −23.31 | −3.64(−3.78-−3.5) | 81.02 | −0.51(−0.78-−0.25) |  |
| Fiji | 117.02 | 0.66(0.39-0.93) | 46.47 | −0.64(−0.85-−0.42) | 123.64 | 0.8(0.59-1.01) |  |
| Finland | 32.1 | −0.38(−0.48-−0.28) | −46.16 | −3.67(−3.86-−3.48) | 31.31 | −0.8(−0.84-−0.76) |  |
| France | −26.05 | −2.86(−2.98-−2.75) | −25.09 | −2.48(−2.57-−2.39) | 8 | −1.51(−1.58-−1.45) |  |
| Gabon | 56.44 | −0.95(−1.09-−0.81) | 10.99 | −1.97(−2.06-−1.87) | 88.76 | −0.16(−0.24-−0.09) |  |
| Gambia | 242.76 | 0.77(0.58-0.96) | 128.1 | −0.61(−0.77-−0.46) | 265.3 | 0.93(0.75-1.1) |  |
| Georgia | −21.43 | 0.49(−0.09-1.08) | −40.8 | −0.53(−0.98-−0.07) | −2.17 | 1.31(0.78-1.85) |  |
| Germany | 51.23 | 0.14(−0.05-0.33) | −28.74 | −2.44(−2.61-−2.27) | 1.43 | −1.6(−2.06-−1.15) |  |
| Ghana | 164.78 | 0.35(0.22-0.48) | 62.33 | −1.55(−1.69-−1.42) | 244.75 | 1.12(1.09-1.15) |  |
| Greece | −9.87 | −1.64(−1.73-−1.55) | −21.44 | −2.33(−2.44-−2.21) | 54.42 | −0.27(−0.41-−0.13) |  |
| Greenland | 54.36 | −0.91(−1.03-−0.78) | −4.64 | −2.43(−2.55-−2.3) | 81.99 | −0.43(−0.69-−0.16) |  |
| Grenada | 60.52 | −0.19(−0.32-−0.06) | −1.79 | −1.57(−1.73-−1.42) | 107.95 | 1(0.86-1.14) |  |
| Guam | 149.58 | 0.61(0.13-1.1) | 72.91 | −1.01(−1.3-−0.71) | 114.65 | −0.14(−0.49-0.21) |  |
| Guatemala | 254.81 | 0.43(−0.05-0.92) | 154.85 | −1(−1.56-−0.43) | 416.55 | 1.98(1.68-2.27) |  |
| Guinea | 95.7 | 0.79(0.68-0.9) | 64.87 | 0.17(0.07-0.27) | 115.2 | 0.9(0.87-0.94) |  |
| Guinea-Bissau | 109.49 | 0.97(0.78-1.16) | 24.35 | −0.92(−1.12-−0.72) | 84.04 | 0.06(0.02-0.1) |  |
| Guyana | 60.98 | −0.08(−0.21-0.04) | −2.14 | −1.73(−1.88-−1.58) | 88.23 | 0.54(0.38-0.71) |  |
| Haiti | 65.74 | −0.71(−0.9-−0.51) | 37.17 | −1.39(−1.56-−1.23) | 114.47 | 0.22(0.12-0.33) |  |
| Honduras | 276.83 | 1.28(1.12-1.45) | 169.97 | 0.15(0.02-0.28) | 291.23 | 1.39(1.29-1.49) |  |
| Hungary | −3.7 | −1.48(−1.9-−1.07) | −47.05 | −3.1(−3.21-−2.98) | 18.66 | −0.41(−0.59-−0.23) |  |
| Iceland | 64.52 | −0.61(−0.75-−0.47) | −36 | −3.85(−3.94-−3.76) | 46.08 | −1.1(−1.23-−0.97) |  |
| India | 96.67 | −0.95(−1.09-−0.8) | 57.68 | −1.45(−1.54-−1.35) | 209.27 | 0.79(0.63-0.94) |  |
| Indonesia | 88.05 | −0.36(−0.4-−0.33) | 34.68 | −1.34(−1.39-−1.29) | 229.57 | 1.62(1.51-1.74) |  |
| Iran  (Islamic Republic of) | 93.11 | −1.21(−1.3-−1.11) | 58.04 | −1.66(−1.87-−1.45) | 226.31 | 0.95(0.78-1.12) |  |
| Iraq | 199.76 | −0.3(−0.36-−0.24) | 145.71 | −0.8(−0.98-−0.63) | 246.7 | 0.64(0.44-0.83) |  |
| Ireland | 52.97 | −0.81(−0.9-−0.72) | −23.8 | −3.14(−3.22-−3.05) | 18.43 | −1.72(−1.79-−1.64) |  |
| Israel | 75.57 | −1.43(−1.58-−1.29) | 28.16 | −2.58(−2.75-−2.4) | 70.28 | −1.89(−2.2-−1.58) |  |
| Italy | −21.21 | −2.34(−2.46-−2.22) | −35.25 | −3.04(−3.13-−2.94) | 17.91 | −1.11(−1.23-−0.99) |  |
| Jamaica | 49.52 | −0.6(−0.96-−0.24) | 16.28 | −1.14(−1.48-−0.8) | 182.47 | 1.92(1.56-2.28) |  |
| Japan | 29.69 | −1.12(−1.29-−0.95) | −27.71 | −3.39(−3.44-−3.33) | 45.56 | −0.91(−0.97-−0.85) |  |
| Jordan | 302.89 | −1.01(−1.13-−0.89) | 174.65 | −2.1(−2.19-−2.01) | 351.03 | −0.03(−0.11-0.05) |  |
| Kazakhstan | −51.53 | −3.75(−3.91-−3.6) | −46.89 | −3.16(−3.31-−3.02) | 6.1 | −0.66(−0.79-−0.52) |  |
| Kenya | 246.76 | 1.21(0.95-1.48) | 183.48 | 0.47(0.19-0.75) | 327.6 | 1.92(1.73-2.12) |  |
| Kiribati | 73.91 | −0.44(−0.58-−0.3) | 52.71 | −0.75(−0.8-−0.69) | 70.82 | −0.42(−0.49-−0.36) |  |
| Kuwait | 182.53 | −1.3(−1.65-−0.95) | 100.68 | −2.35(−2.69-−2.01) | 373 | 1.51(1.01-2.02) |  |
| Kyrgyzstan | −29.06 | −2.99(−3.2-−2.77) | −22.61 | −2.63(−2.92-−2.33) | −9.04 | −2.01(−2.24-−1.79) |  |
| Lao People's Democratic Republic | 19.66 | −2.21(−2.35-−2.07) | −10.09 | −3.14(−3.25-−3.03) | 150.92 | 0.47(0.41-0.53) |  |
| Latvia | 3.99 | 0.34(0.05-0.64) | −57.78 | −3.12(−3.41-−2.83) | −6.8 | −0.58(−0.87-−0.3) |  |
| Lebanon | 91.59 | −0.28(−0.48-−0.08) | 40.87 | −1.38(−1.5-−1.25) | 181.87 | 1.28(1.1-1.45) |  |
| Lesotho | 67.31 | 1.12(0.86-1.38) | 62.14 | 1.24(0.95-1.54) | 151 | 2.69(2.46-2.92) |  |
| Liberia | 148.14 | 1.38(1.05-1.72) | 34.46 | −1.07(−1.28-−0.86) | 83.45 | 0.24(−0.12-0.6) |  |
| Libya | 212.86 | 0.38(0.26-0.5) | 107.82 | −0.97(−1.14-−0.81) | 188.63 | 0.3(0.15-0.46) |  |
| Lithuania | 30.76 | 0.85(0.53-1.16) | −49.6 | −2.71(−2.94-−2.48) | 10.8 | −0.32(−0.55-−0.09) |  |
| Luxembourg | 18.13 | −1.63(−1.75-−1.51) | −34.96 | −3.75(−3.83-−3.68) | 1.57 | −2.2(−2.37-−2.04) |  |
| Madagascar | 80 | −1(−1.15-−0.85) | 62.5 | −1.2(−1.29-−1.1) | 125.98 | 0.03(−0.02-0.09) |  |
| Malawi | 106.98 | 0.42(0.07-0.77) | 29.52 | −1.62(−1.83-−1.42) | 134.62 | 0.85(0.68-1.01) |  |
| Malaysia | 152.4 | −0.87(−1.07-−0.68) | 71.02 | −2.02(−2.18-−1.86) | 225.65 | 0.05(−0.16-0.26) |  |
| Maldives | 27.77 | −3.78(−4.06-−3.49) | −4.02 | −4.87(−5.19-−4.56) | 169.87 | −1.22(−1.44-−1.01) |  |
| Mali | 105.26 | −0.04(−0.11-0.02) | 56.8 | −0.95(−1.06-−0.84) | 142.24 | 0.47(0.4-0.54) |  |
| Malta | 44.82 | −0.93(−0.98-−0.88) | −18.76 | −3.02(−3.1-−2.94) | 66.83 | −0.65(−0.75-−0.54) |  |
| Marshall Islands | 98.28 | −0.41(−0.48-−0.33) | 71.55 | −0.67(−0.76-−0.58) | 136.74 | 0.42(0.38-0.46) |  |
| Mauritania | 87.83 | 0.16(−0.07-0.4) | 9.6 | −1.74(−2.03-−1.46) | 77.44 | −0.33(−0.5-−0.16) |  |
| Mauritius | 70.76 | −1.21(−1.35-−1.07) | 5.08 | −2.7(−2.81-−2.58) | 241.22 | 1.46(1.31-1.62) |  |
| Mexico | 102.98 | −1.1(−1.24-−0.96) | 76.05 | −1.61(−1.73-−1.5) | 285.67 | 1.51(1.42-1.59) |  |
| Micronesia  (Federated States of) | 37.26 | −0.62(−0.67-−0.56) | 17.84 | −0.78(−0.87-−0.7) | 64.74 | 0.3(0.27-0.34) |  |
| Monaco | 35.69 | 0.15(0.04-0.26) | −25.13 | −2.01(−2.23-−1.79) | 39.26 | 0.19(0.11-0.26) |  |
| Mongolia | 86.81 | −0.96(−1.2-−0.71) | 50.5 | −2.21(−2.47-−1.95) | 127.77 | −0.59(−0.77-−0.4) |  |
| Montenegro | 63.12 | 0.42(0.23-0.61) | 22.57 | −0.86(−1.13-−0.58) | 77.53 | 0.75(0.65-0.84) |  |
| Morocco | 139.6 | 0.1(−0.02-0.23) | 74.95 | −0.87(−0.98-−0.76) | 191.22 | 0.88(0.77-0.98) |  |
| Mozambique | 136.3 | 1.25(1.1-1.39) | 91.71 | 0.47(0.33-0.61) | 221.35 | 2.33(2.16-2.5) |  |
| Myanmar | 18.67 | −1.86(−1.93-−1.79) | −20.07 | −3.18(−3.3-−3.07) | 150.31 | 1.08(0.98-1.18) |  |
| Namibia | 113.33 | −0.04(−0.36-0.27) | 55.7 | −0.98(−1.26-−0.71) | 179.43 | 1.32(1.16-1.48) |  |
| Nauru | −0.21 | −0.87(−1.19-−0.55) | −2.49 | −0.67(−1.02-−0.31) | 3.97 | −0.52(−0.72-−0.32) |  |
| Nepal | 94.28 | −0.3(−0.68-0.08) | 69.64 | −0.67(−0.95-−0.39) | 189.14 | 1.02(0.77-1.27) |  |
| Netherlands | 162 | 1.53(1.17-1.89) | −15.77 | −2.57(−2.71-−2.43) | 48.79 | −0.52(−0.61-−0.42) |  |
| New Zealand | 60.37 | −0.84(−0.92-−0.76) | −6.81 | −2.61(−2.73-−2.49) | 20.64 | −1.88(−2-−1.75) |  |
| Nicaragua | 172.45 | −0.63(−0.84-−0.42) | 120.94 | −1.02(−1.25-−0.79) | 313.08 | 1.73(1.55-1.91) |  |
| Niger | 205.43 | 0.79(0.62-0.97) | 107.46 | −0.6(−0.79-−0.4) | 163.76 | −0.08(−0.2-0.05) |  |
| Nigeria | 95.18 | 0.08(−0.08-0.23) | 57.82 | −0.52(−0.64-−0.4) | 158.42 | 1.28(1.1-1.45) |  |
| Niue | −6.56 | −0.58(−0.72-−0.44) | −28.16 | −1.34(−1.43-−1.25) | 2.5 | 0.05(0.02-0.09) |  |
| North Macedonia | 64.27 | −0.09(−0.27-0.09) | 2.39 | −2.03(−2.17-−1.89) | 126.1 | 1.15(0.91-1.39) |  |
| Northern Mariana Islands | 384.85 | 3.04(2.6-3.48) | 28.72 | −1.82(−2.07-−1.57) | 113.21 | −0.33(−0.46-−0.19) |  |
| Norway | 34.31 | −0.15(−0.25-−0.06) | −44.81 | −3.37(−3.43-−3.31) | 14.92 | −0.78(−0.92-−0.63) |  |
| Oman | 131.29 | −0.18(−0.49-0.14) | 23.65 | −2.06(−2.34-−1.78) | 191.45 | 0.86(0.54-1.18) |  |
| Pakistan | 131.86 | 0.22(−0.02-0.46) | 103.63 | −0.2(−0.47-0.08) | 220.63 | 1.42(1.15-1.7) |  |
| Palau | 129.19 | −0.07(−0.19-0.05) | 65.21 | −0.67(−0.72-−0.63) | 102.75 | −0.13(−0.21-−0.06) |  |
| Palestine | 115.76 | −1.17(−1.28-−1.05) | 67.06 | −1.84(−2.04-−1.65) | 244.8 | 0.88(0.64-1.11) |  |
| Panama | 151.83 | 0.12(−0.13-0.36) | 55.9 | −1.9(−2.1-−1.7) | 222.63 | 0.95(0.85-1.04) |  |
| Papua New Guinea | 154.07 | −0.1(−0.15-−0.04) | 154.4 | −0.06(−0.15-0.03) | 203.2 | 0.46(0.42-0.49) |  |
| Paraguay | 204.51 | 0.46(0.3-0.62) | 98.22 | −1.4(−1.82-−0.98) | 370.55 | 2.56(2.35-2.76) |  |
| Peru | 76.56 | −1.31(−1.46-−1.17) | 59.86 | −1.64(−1.87-−1.41) | 186.61 | 0.62(0.4-0.84) |  |
| Philippines | 112.41 | −0.85(−1.02-−0.68) | 47.73 | −1.94(−2.12-−1.76) | 182.62 | 0.33(0.08-0.58) |  |
| Poland | 29.82 | −0.66(−0.76-−0.55) | −29.93 | −2.73(−2.82-−2.64) | 66.65 | 0.11(−0.03-0.26) |  |
| Portugal | 3.53 | −1.02(−1.21-−0.82) | −34.45 | −3.12(−3.19-−3.05) | 45.67 | −0.52(−0.71-−0.33) |  |
| Puerto Rico | −32.09 | −3.76(−4.02-−3.5) | −24.86 | −3.38(−3.55-−3.22) | 101.46 | 0.38(0.17-0.59) |  |
| Qatar | 448.83 | −0.91(−1.2-−0.63) | 260.09 | −2.19(−2.51-−1.88) | 629.87 | 0.55(0.24-0.86) |  |
| Republic of Korea | 0.97 | −4.4(−4.74-−4.06) | −46.66 | −5.88(−6.07-−5.69) | 163.34 | −0.46(−0.73-−0.19) |  |
| Republic of Moldova | −12.7 | −1(−1.46-−0.53) | −45 | −2.08(−2.49-−1.67) | 14.34 | 0.52(−0.02-1.05) |  |
| Romania | 89.47 | 1.51(1.22-1.81) | −20.02 | −1.59(−1.76-−1.42) | 76.02 | 1.02(0.81-1.23) |  |
| Russian Federation | −15.41 | −1.72(−1.97-−1.47) | −47.75 | −3.41(−3.71-−3.11) | 28.43 | −0.28(−0.59-0.04) |  |
| Rwanda | 15.27 | −3.01(−3.38-−2.64) | −1.56 | −3.54(−3.92-−3.15) | 89.21 | −0.77(−1.03-−0.51) |  |
| Saint Kitts and Nevis | 71.68 | −1.11(−1.43-−0.8) | −3.73 | −2.66(−2.91-−2.4) | 73.24 | −0.34(−0.51-−0.17) |  |
| Saint Lucia | 111.23 | −0.75(−1.04-−0.46) | 36.86 | −2.31(−2.62-−2) | 141.25 | −0.3(−0.55-−0.04) |  |
| Saint Vincent and  the Grenadines | 109.29 | 0.22(−0.07-0.52) | 25.55 | −1.56(−1.81-−1.32) | 127.81 | 0.57(0.39-0.75) |  |
| Samoa | 30.64 | −1.08(−1.16-−1.01) | 25.53 | −0.97(−1.05-−0.9) | 59.28 | −0.21(−0.25-−0.17) |  |
| San Marino | 64.7 | −0.21(−0.32-−0.1) | 19.6 | −1.58(−1.76-−1.41) | 68.8 | −0.21(−0.29-−0.13) |  |
| Sao Tome and Principe | 179.06 | 2.07(1.96-2.18) | 60.09 | −0.19(−0.33-−0.05) | 154.41 | 1.46(1.39-1.53) |  |
| Saudi Arabia | 212.21 | −0.84(−1.07-−0.61) | 83.64 | −2.46(−2.59-−2.34) | 411.05 | 1.24(1.03-1.45) |  |
| Senegal | 185.44 | 1.29(0.97-1.61) | 71.38 | −0.61(−0.89-−0.33) | 150.27 | 0.45(0.26-0.64) |  |
| Serbia | 20.88 | 0.01(−0.17-0.19) | −23.38 | −2.15(−2.38-−1.93) | 48.99 | 0.48(0.33-0.63) |  |
| Seychelles | 101.75 | −0.47(−0.6-−0.34) | 14.4 | −2.3(−2.45-−2.16) | 186.22 | 0.86(0.63-1.1) |  |
| Sierra Leone | 151.19 | 1.45(1.21-1.68) | 56.04 | −0.27(−0.48-−0.06) | 115.27 | 0.56(0.39-0.72) |  |
| Singapore | 8.16 | −4.31(−4.6-−4.03) | −21.6 | −5.25(−5.45-−5.06) | 82.1 | −2.45(−2.57-−2.34) |  |
| Slovakia | 26.38 | −0.78(−0.99-−0.58) | −35.08 | −2.77(−2.95-−2.58) | 53.18 | −0.05(−0.21-0.11) |  |
| Slovenia | 0.32 | −1.9(−2.06-−1.73) | −35.2 | −3.48(−3.6-−3.36) | 43.85 | −0.82(−1.12-−0.52) |  |
| Solomon Islands | 119.75 | −0.24(−0.36-−0.12) | 118.02 | −0.23(−0.29-−0.16) | 173.86 | 0.51(0.42-0.61) |  |
| Somalia | 89.91 | −1.03(−1.11-−0.96) | 101.3 | −0.91(−0.95-−0.87) | 146.94 | −0.05(−0.13-0.03) |  |
| South Africa | 37.68 | −2.11(−2.75-−1.47) | 22.68 | −1.98(−2.4-−1.55) | 115.44 | 0.24(0.01-0.47) |  |
| South Sudan | 21.39 | −1.33(−1.39-−1.26) | 14.98 | −1.33(−1.39-−1.28) | 58.17 | −0.11(−0.21-−0.01) |  |
| Spain | −0.23 | −1.98(−2.08-−1.88) | −27.74 | −2.87(−3.11-−2.62) | 60.33 | −0.25(−0.3-−0.2) |  |
| Sri Lanka | 95.61 | −0.01(−0.43-0.41) | 15.71 | −2.12(−2.3-−1.95) | 170.39 | 1.03(0.93-1.13) |  |
| Sudan | 113.49 | 0.16(0.06-0.27) | 66.68 | −0.59(−0.66-−0.52) | 178.47 | 1.15(1.06-1.24) |  |
| Suriname | 135.67 | 0(−0.29-0.29) | 58.15 | −1.51(−1.82-−1.2) | 194.83 | 0.98(0.75-1.2) |  |
| Sweden | 40.11 | 0.04(−0.09-0.18) | −50.09 | −3.55(−3.7-−3.4) | 15.18 | −0.69(−0.83-−0.56) |  |
| Switzerland | 7.32 | −1.36(−1.49-−1.24) | −42.01 | −3.43(−3.8-−3.06) | 30.27 | −1.09(−1.27-−0.91) |  |
| Syrian Arab Republic | 121.6 | −0.42(−0.64-−0.19) | 57.34 | −1.19(−1.46-−0.91) | 111.07 | −0.08(−0.32-0.16) |  |
| Taiwan  (Province of China) | 289.57 | 2.56(2.29-2.82) | 21.85 | −2.21(−2.35-−2.08) | 240.64 | 1.5(1.3-1.7) |  |
| Tajikistan | 2.63 | −1.99(−2.44-−1.55) | 29.92 | −0.92(−1.2-−0.64) | 53 | −0.11(−0.55-0.34) |  |
| Thailand | 101.47 | −1.55(−1.84-−1.27) | 14.46 | −3.29(−3.57-−3.01) | 141.29 | −0.86(−1.19-−0.52) |  |
| Timor-Leste | 114.57 | −0.61(−0.92-−0.31) | 59.1 | −1.6(−1.9-−1.29) | 284.02 | 1.91(1.65-2.18) |  |
| Togo | 292.93 | 1.28(1.13-1.43) | 127.93 | −0.52(−0.65-−0.39) | 209.99 | 0.21(0.16-0.25) |  |
| Tokelau | −18.23 | −0.82(−0.85-−0.78) | −31.84 | −1.37(−1.4-−1.34) | 5.99 | 0.21(0.18-0.25) |  |
| Tonga | 39.46 | −0.26(−0.58-0.07) | 16.85 | −0.66(−0.82-−0.5) | 56.51 | 0.28(0.16-0.4) |  |
| Trinidad and Tobago | 40.49 | −2.02(−2.36-−1.68) | −18.82 | −3.88(−4.2-−3.56) | 100.15 | −0.41(−0.54-−0.27) |  |
| Tunisia | 150.43 | −0.07(−0.13-−0.01) | 75.94 | −1.24(−1.29-−1.19) | 192.1 | 0.51(0.44-0.59) |  |
| Turkey | 46.76 | −1.65(−1.87-−1.42) | 8 | −2.36(−2.97-−1.74) | 104.48 | −0.25(−0.71-0.21) |  |
| Turkmenistan | −37.99 | −4.47(−5.22-−3.72) | −16.63 | −3.46(−3.84-−3.08) | 49.44 | −1.05(−1.59-−0.5) |  |
| Tuvalu | 12.48 | −1.04(−1.12-−0.97) | 2.04 | −1.12(−1.19-−1.06) | 50.57 | 0(−0.04-0.04) |  |
| Uganda | 173.77 | 0.57(0.37-0.78) | 95.3 | −1.12(−1.44-−0.8) | 300.07 | 1.83(1.58-2.08) |  |
| Ukraine | −15.43 | −1.4(−1.87-−0.94) | −51.82 | −3.59(−4.06-−3.12) | −10.12 | −1.33(−1.7-−0.96) |  |
| United Arab Emirates | 1128.46 | 0.21(−0.09-0.52) | 480.44 | −2.1(−2.48-−1.71) | 767.53 | −0.46(−0.82-−0.09) |  |
| United Kingdom | 39.39 | −0.16(−0.33-0) | −39.9 | −3.09(−3.27-−2.9) | −4.68 | −1.52(−1.65-−1.38) |  |
| United Republic of Tanzania | 78.06 | −0.98(−1.05-−0.92) | 64.57 | −1.18(−1.22-−1.15) | 174.71 | 0.7(0.6-0.79) |  |
| United States of America | 76.35 | −0.17(−0.27-−0.06) | −1.08 | −1.92(−2.04-−1.79) | 27.04 | −1.13(−1.24-−1.02) |  |
| United States Virgin Islands | 137.98 | 1.23(0.89-1.56) | 68.66 | −0.27(−0.49-−0.05) | 167.12 | 1.24(1.04-1.44) |  |
| Uruguay | −17.54 | −1.88(−2.01-−1.75) | −21.58 | −1.82(−1.88-−1.76) | 23.09 | −0.42(−0.49-−0.35) |  |
| Uzbekistan | −23.21 | −4.26(−4.71-−3.8) | 14.09 | −2.34(−2.52-−2.16) | 92.05 | 0.12(−0.06-0.3) |  |
| Vanuatu | 160.07 | −0.25(−0.4-−0.11) | 141.35 | −0.46(−0.59-−0.33) | 211.01 | 0.48(0.32-0.64) |  |
| Venezuela  (Bolivarian Republic of) | 137.83 | −0.88(−1.06-−0.7) | 83.38 | −1.96(−2.15-−1.78) | 265.92 | 0.62(0.48-0.76) |  |
| Viet Nam | 206.93 | 1.19(1.01-1.37) | 24.51 | −2.63(−2.77-−2.49) | 351.64 | 2.69(2.55-2.83) |  |
| Yemen | 153.28 | −0.26(−0.3-−0.22) | 142.13 | −0.47(−0.54-−0.39) | 228.39 | 0.93(0.8-1.06) |  |
| Zambia | 90.41 | −1.25(−1.52-−0.97) | 74.78 | −1.55(−1.85-−1.26) | 194.8 | 0.56(0.52-0.6) |  |
| Zimbabwe | 89.44 | 0.22(−0.03-0.48) | 95.41 | 1(0.7-1.31) | 140.14 | 1.19(0.93-1.44) |  |

YLLs: years of life lost; EAPC: estimated annual percentage change; ASR, age-standardized rate; CI, confidence interval; UI: uncertainty interval. Percentage change in number was calculated based on the crew data.

**Supplementary table 4**. The percentage change in number and EAPCs of YLDs at national level and both sexes from 1990 to 2019

| **Characteristics** | **Esophageal cancer** | | **Stomach cancer** | | **Colorectal cancer** | | |
| --- | --- | --- | --- | --- | --- | --- | --- |
|  | Percentage change in  number (%) | EAPC  (95%CI) | Percentage change in  number (%) | EAPC  (95%CI) | Percentage change in  number (%) | EAPC  (95%CI) | |
| Afghanistan | 63.61 | −0.41(−0.53-−0.29) | 75.2 | −0.39(−0.59-−0.2) | 118.65 | 0.53(0.31-0.75) |  |
| Albania | 38.47 | −1.01(−1.33-−0.68) | 52.51 | −0.86(−1.05-−0.66) | 201.73 | 2.06(1.8-2.31) |  |
| Algeria | 136.79 | −0.71(−0.87-−0.56) | 72.9 | −1.73(−1.88-−1.58) | 302.15 | 1.24(1.2-1.29) |  |
| American Samoa | 142.19 | 0.56(0.39-0.73) | 63.45 | −0.63(−0.78-−0.49) | 133.94 | 0.6(0.51-0.69) |  |
| Andorra | 153.69 | −0.04(−0.14-0.06) | 133.1 | −0.38(−0.42-−0.33) | 189.75 | 0.48(0.33-0.63) |  |
| Angola | 107.99 | −1.3(−1.41-−1.19) | 73.56 | −1.75(−1.82-−1.68) | 270.95 | 1(0.8-1.2) |  |
| Antigua and Barbuda | 81.37 | −0.28(−0.51-−0.05) | 33.87 | −1.22(−1.41-−1.02) | 213.67 | 1.71(1.59-1.83) |  |
| Argentina | 17.32 | −1.52(−1.67-−1.36) | 22.6 | −1.13(−1.22-−1.04) | 149.12 | 1.26(1.11-1.41) |  |
| Armenia | 2.63 | −1.31(−1.61-−1.01) | −13.19 | −1.86(−2.04-−1.67) | 80.52 | 0.83(0.71-0.95) |  |
| Australia | 104.23 | −0.27(−0.39-−0.14) | 66.14 | −0.86(−0.93-−0.79) | 113.38 | −0.16(−0.32-0) |  |
| Austria | 36.62 | −0.47(−0.64-−0.31) | −28.35 | −2.89(−3.06-−2.72) | 8.76 | −1.59(−1.76-−1.42) |  |
| Azerbaijan | 57.38 | −0.55(−0.74-−0.35) | 25.56 | −1.19(−1.39-−0.98) | 132.28 | 1.28(0.75-1.81) |  |
| Bahamas | 101.87 | −0.62(−0.79-−0.44) | 68.8 | −1.37(−1.48-−1.26) | 213.36 | 1.09(0.95-1.22) |  |
| Bahrain | 172.4 | −3.25(−3.56-−2.94) | 160.47 | −2.55(−2.79-−2.31) | 666.21 | 0.98(0.76-1.2) |  |
| Bangladesh | 81.06 | −1.29(−1.41-−1.18) | 57.84 | −1.67(−1.83-−1.5) | 251.02 | 0.81(0.69-0.93) |  |
| Barbados | 54.59 | −0.65(−0.76-−0.54) | 16.93 | −1.52(−1.66-−1.38) | 201.37 | 2.01(1.78-2.23) |  |
| Belarus | 21.45 | −0.49(−0.66-−0.32) | −33.91 | −2.65(−2.87-−2.43) | 71.9 | 0.68(0.42-0.95) |  |
| Belgium | 98.7 | 1.13(0.93-1.34) | −9.18 | −1.48(−1.64-−1.31) | 48.73 | 0.03(−0.02-0.09) |  |
| Belize | 281.6 | 0.6(0.25-0.95) | 164.93 | −0.8(−1.25-−0.35) | 456.61 | 1.9(1.5-2.29) |  |
| Benin | 225.39 | 1.37(1.19-1.56) | 85.91 | −0.57(−0.71-−0.44) | 199.43 | 0.78(0.74-0.82) |  |
| Bermuda | 34.36 | −1.2(−1.45-−0.95) | 21.27 | −1.76(−1.91-−1.61) | 146.59 | 0.73(0.66-0.79) |  |
| Bhutan | 87.26 | −0.5(−0.64-−0.36) | 82.1 | −0.46(−0.53-−0.38) | 313.94 | 2.22(2.16-2.28) |  |
| Bolivia  (Plurinational State of) | 146.98 | −0.45(−0.55-−0.34) | 101.45 | −1.11(−1.22-−1.01) | 417.75 | 2.09(2-2.19) |  |
| Bosnia and Herzegovina | 17.44 | −0.95(−1.07-−0.82) | 13.7 | −0.84(−1.03-−0.66) | 189.42 | 3.24(2.89-3.6) |  |
| Botswana | 126.85 | −0.89(−1.3-−0.47) | 92.54 | −1.28(−1.64-−0.92) | 335.87 | 1.64(1.39-1.89) |  |
| Brazil | 108.69 | −0.77(−0.83-−0.72) | 59.11 | −1.8(−1.87-−1.73) | 329.27 | 1.79(1.56-2.01) |  |
| Brunei Darussalam | 116.34 | −1.42(−1.63-−1.2) | 49.45 | −2.33(−2.55-−2.12) | 287.85 | 1.5(1.26-1.75) |  |
| Bulgaria | −14.54 | −0.06(−0.7-0.59) | −29.31 | −0.77(−1.11-−0.42) | 80.83 | 2.93(2.48-3.39) |  |
| Burkina Faso | 171.46 | 1.2(1.01-1.39) | 77.14 | −0.39(−0.63-−0.14) | 178.19 | 0.93(0.79-1.07) |  |
| Burundi | 37.8 | −1.71(−1.87-−1.55) | 32.81 | −1.68(−1.8-−1.56) | 88.16 | −0.29(−0.37-−0.22) |  |
| Cabo Verde | 212.19 | 0.94(0.6-1.29) | 42.21 | −1.42(−1.9-−0.94) | 407.68 | 3.11(2.9-3.31) |  |
| Cambodia | 110.41 | −0.87(−1.02-−0.73) | 52.5 | −1.86(−1.97-−1.74) | 384.55 | 2.26(2.17-2.35) |  |
| Cameroon | 254.13 | 1.28(1.12-1.45) | 120.15 | −0.4(−0.51-−0.3) | 244.87 | 0.87(0.82-0.91) |  |
| Canada | 161.34 | 0.81(0.72-0.9) | 76.8 | −0.5(−0.55-−0.44) | 135.08 | 0.48(0.38-0.58) |  |
| Central African Republic | 45.24 | −0.95(−1.04-−0.87) | 39.73 | −1.06(−1.11-−1.02) | 66.47 | −0.32(−0.38-−0.27) |  |
| Chad | 185.55 | 1.63(1.47-1.78) | 89.86 | 0.17(0.08-0.26) | 177.72 | 1.24(1.18-1.29) |  |
| Chile | 36.98 | −2.58(−2.8-−2.37) | 54.71 | −1.48(−1.57-−1.38) | 335.6 | 2.47(2.33-2.6) |  |
| China | 71.27 | −1.31(−1.74-−0.87) | 128.84 | 0.26(−0.04-0.57) | 555.15 | 4.4(4.07-4.72) |  |
| Colombia | 53.3 | −2.86(−3.06-−2.65) | 72.09 | −2.13(−2.27-−1.98) | 389.09 | 1.73(1.64-1.82) |  |
| Comoros | 74.89 | −1.1(−1.29-−0.91) | 59.16 | −1.31(−1.46-−1.15) | 164.59 | 0.52(0.4-0.63) |  |
| Congo | 68.21 | −1.54(−1.71-−1.36) | 36.74 | −2.14(−2.28-−1.99) | 154.66 | 0.1(−0.1-0.3) |  |
| Cook Islands | 67.2 | −0.75(−0.85-−0.64) | 31.21 | −1.16(−1.34-−0.98) | 124.94 | 0.49(0.29-0.69) |  |
| Costa Rica | 94.18 | −1.83(−2.07-−1.59) | 85.94 | −1.96(−2.22-−1.7) | 508.31 | 2.9(2.79-3) |  |
| Croatia | −7.07 | −1.01(−1.13-−0.89) | −24.69 | −1.89(−2.01-−1.77) | 108.63 | 1.96(1.79-2.13) |  |
| Cuba | 162.79 | 1.67(1.48-1.86) | 62.01 | −0.21(−0.32-−0.11) | 189.9 | 1.67(1.57-1.77) |  |
| Cyprus | 204.67 | 1.07(0.76-1.39) | 189.9 | 1.03(0.72-1.35) | 396.42 | 3.04(2.67-3.41) |  |
| Czechia | 73.14 | 0.63(0.48-0.77) | −25.03 | −2.44(−2.53-−2.35) | 47.58 | −0.36(−0.61-−0.1) |  |
| Cote d'Ivoire | 229.17 | 0.99(0.87-1.11) | 93.54 | −0.86(−0.93-−0.79) | 183.6 | 0.08(−0.01-0.17) |  |
| Democratic People's Republic of Korea | 74.3 | −0.38(−0.43-−0.32) | 45.52 | −0.92(−1.01-−0.84) | 112.78 | 0.53(0.35-0.72) |  |
| Democratic Republic of  the Congo | 79.13 | −0.94(−1.02-−0.86) | 61.82 | −1.18(−1.22-−1.13) | 125.12 | −0.22(−0.53-0.1) |  |
| Denmark | 52.25 | 0.29(0.13-0.44) | −12.24 | −1.3(−1.53-−1.07) | 95.27 | 1.07(0.86-1.28) |  |
| Djibouti | 266.52 | −0.68(−0.8-−0.56) | 230.08 | −0.85(−0.96-−0.75) | 472.32 | 1.14(1.04-1.23) |  |
| Dominica | 28.33 | 0.08(−0.07-0.23) | −10.17 | −1.15(−1.26-−1.04) | 61.41 | 1.03(0.92-1.14) |  |
| Dominican Republic | 267.48 | 1.89(1.71-2.07) | 195.99 | 1.07(0.92-1.22) | 438.2 | 3.19(3.08-3.31) |  |
| Ecuador | 110.3 | −0.9(−1.1-−0.69) | 114.87 | −0.84(−1.05-−0.62) | 587.12 | 3.49(3.25-3.73) |  |
| Egypt | 170.75 | 0.85(0.74-0.96) | 93.47 | −0.18(−0.28-−0.08) | 286.21 | 1.88(1.79-1.97) |  |
| El Salvador | 130 | 0.04(−0.33-0.4) | 116.47 | −0.22(−0.58-0.15) | 424.97 | 3.33(2.9-3.76) |  |
| Equatorial Guinea | 66.01 | −1.25(−1.51-−1) | 6.02 | −2.98(−3.29-−2.67) | 511.43 | 4.01(3.71-4.31) |  |
| Eritrea | 129.48 | −0.62(−0.82-−0.41) | 119.42 | −0.63(−0.75-−0.51) | 320.51 | 1.35(1.11-1.6) |  |
| Estonia | 9.49 | −0.15(−0.39-0.09) | −25.69 | −1.57(−1.76-−1.37) | 90 | 1.8(1.59-2.01) |  |
| Eswatini | 87.17 | −0.38(−0.94-0.18) | 65.48 | −0.38(−0.76-0) | 179.91 | 1.19(0.93-1.45) |  |
| Ethiopia | 18.84 | −1.92(−2.05-−1.8) | −13.66 | −3.11(−3.23-−2.98) | 120.4 | 0.18(−0.1-0.47) |  |
| Fiji | 128.48 | 0.67(0.42-0.93) | 60.48 | −0.54(−0.73-−0.35) | 154.25 | 1.02(0.82-1.22) |  |
| Finland | 70.83 | 0.46(0.36-0.56) | −17.44 | −2.37(−2.52-−2.23) | 116.44 | 0.98(0.91-1.04) |  |
| France | 3.36 | −1.67(−1.75-−1.59) | 14.37 | −1(−1.07-−0.93) | 76.75 | 0.39(0.27-0.51) |  |
| Gabon | 58.65 | −0.77(−0.88-−0.66) | 16.33 | −1.68(−1.75-−1.61) | 117.83 | 0.38(0.33-0.43) |  |
| Gambia | 249.52 | 0.79(0.62-0.96) | 139.55 | −0.5(−0.64-−0.37) | 291.31 | 1.08(0.94-1.22) |  |
| Georgia | −16.68 | 0.54(−0.04-1.13) | −35.06 | −0.44(−0.86-−0.01) | 8.44 | 1.4(1-1.8) |  |
| Germany | 140.66 | 1.67(1.3-2.05) | −2.43 | −1.61(−1.76-−1.45) | 50.78 | −0.36(−0.82-0.09) |  |
| Ghana | 167.99 | 0.34(0.23-0.45) | 73.6 | −1.34(−1.48-−1.2) | 290.56 | 1.43(1.39-1.47) |  |
| Greece | 9.73 | −1.31(−1.4-−1.21) | 5.75 | −1.48(−1.6-−1.36) | 103.14 | 0.77(0.57-0.97) |  |
| Greenland | 74.28 | −0.65(−0.8-−0.5) | 16.82 | −1.91(−2.05-−1.76) | 161.61 | 0.67(0.42-0.91) |  |
| Grenada | 54.57 | −0.17(−0.28-−0.06) | 4.27 | −1.21(−1.33-−1.1) | 163.46 | 1.78(1.69-1.87) |  |
| Guam | 162.93 | 0.39(−0.02-0.8) | 92.05 | −1.09(−1.33-−0.84) | 145.43 | 0.02(−0.17-0.21) |  |
| Guatemala | 281.69 | 0.48(0.01-0.95) | 193.19 | −0.67(−1.25-−0.09) | 582.41 | 2.85(2.5-3.21) |  |
| Guinea | 93.08 | 0.77(0.67-0.88) | 63.41 | 0.18(0.09-0.26) | 122.95 | 1.02(1-1.05) |  |
| Guinea-Bissau | 107.03 | 0.98(0.79-1.16) | 26.45 | −0.74(−0.94-−0.53) | 95.99 | 0.37(0.32-0.42) |  |
| Guyana | 59.95 | −0.17(−0.29-−0.04) | 3.4 | −1.62(−1.77-−1.48) | 121.56 | 0.87(0.69-1.05) |  |
| Haiti | 70.6 | −0.62(−0.77-−0.46) | 42.97 | −1.23(−1.37-−1.09) | 144.29 | 0.59(0.48-0.7) |  |
| Honduras | 304.81 | 1.5(1.33-1.68) | 216.06 | 0.6(0.46-0.75) | 424.51 | 2.2(2.11-2.29) |  |
| Hungary | 9.71 | −0.98(−1.37-−0.59) | −31.54 | −2.35(−2.46-−2.24) | 78.41 | 1(0.75-1.25) |  |
| Iceland | 100.87 | 0.04(−0.02-0.11) | −7.15 | −2.6(−2.71-−2.49) | 107.61 | 0.14(−0.12-0.41) |  |
| India | 111.23 | −0.84(−0.98-−0.7) | 74.31 | −1.28(−1.38-−1.19) | 291.94 | 1.5(1.34-1.66) |  |
| Indonesia | 103.08 | −0.13(−0.17-−0.09) | 53.78 | −0.92(−0.97-−0.88) | 313.63 | 2.32(2.26-2.38) |  |
| Iran  (Islamic Republic of) | 127.11 | −0.79(−0.88-−0.69) | 102.02 | −1.03(−1.24-−0.82) | 391.82 | 2.06(1.9-2.21) |  |
| Iraq | 210.56 | −0.07(−0.15-0.01) | 170.46 | −0.38(−0.58-−0.18) | 370.14 | 1.74(1.45-2.04) |  |
| Ireland | 106.77 | 0.37(0.29-0.46) | 30.27 | −1.09(−1.17-−1.01) | 114.99 | 0.55(0.46-0.63) |  |
| Israel | 120.48 | −0.71(−0.83-−0.58) | 85.39 | −1.35(−1.52-−1.19) | 175.15 | −0.09(−0.43-0.25) |  |
| Italy | 6.2 | −1.43(−1.5-−1.37) | 6 | −1.38(−1.45-−1.3) | 87.93 | 0.63(0.37-0.89) |  |
| Jamaica | 47.47 | −0.55(−0.85-−0.25) | 21.66 | −0.91(−1.21-−0.62) | 222.86 | 2.49(2.19-2.79) |  |
| Japan | 109.27 | 0.44(0.18-0.7) | −5.97 | −2.64(−2.7-−2.58) | 125.21 | 0.47(0.39-0.56) |  |
| Jordan | 344.79 | −0.64(−0.75-−0.53) | 243.66 | −1.29(−1.38-−1.2) | 641.62 | 1.72(1.59-1.85) |  |
| Kazakhstan | −47.92 | −3.48(−3.64-−3.31) | −38.79 | −2.64(−2.78-−2.5) | 46.64 | 0.59(0.36-0.81) |  |
| Kenya | 220.96 | 0.88(0.67-1.09) | 164.74 | 0.16(−0.05-0.38) | 322.73 | 1.75(1.62-1.89) |  |
| Kiribati | 75.67 | −0.31(−0.43-−0.18) | 57.45 | −0.58(−0.64-−0.53) | 86.69 | −0.1(−0.16-−0.03) |  |
| Kuwait | 235.7 | −0.74(−1.05-−0.42) | 174.37 | −1.26(−1.54-−0.99) | 645.06 | 2.84(2.53-3.15) |  |
| Kyrgyzstan | −23.97 | −2.57(−2.77-−2.38) | −16.17 | −2.18(−2.49-−1.88) | 16.85 | −1.01(−1.28-−0.73) |  |
| Lao People's Democratic Republic | 27.56 | −1.92(−2.06-−1.78) | 0.82 | −2.67(−2.78-−2.56) | 210.29 | 1.23(1.13-1.32) |  |
| Latvia | 11.35 | 0.53(0.27-0.79) | −39.91 | −1.92(−2.19-−1.66) | 39.9 | 1.06(0.85-1.28) |  |
| Lebanon | 144.92 | 0.43(0.22-0.64) | 110.6 | −0.08(−0.25-0.09) | 419.71 | 3.55(3.32-3.77) |  |
| Lesotho | 58.88 | 0.93(0.73-1.13) | 52.71 | 1.02(0.79-1.25) | 137.89 | 2.4(2.26-2.54) |  |
| Liberia | 136.51 | 1.38(1.06-1.7) | 34.03 | −0.86(−1.06-−0.67) | 97.48 | 0.68(0.35-1.02) |  |
| Libya | 219.4 | 0.55(0.43-0.66) | 125.73 | −0.57(−0.72-−0.42) | 263 | 1.21(0.99-1.43) |  |
| Lithuania | 41.18 | 1.02(0.74-1.3) | −32.56 | −1.84(−2.02-−1.65) | 52.83 | 0.88(0.72-1.04) |  |
| Luxembourg | 53.86 | −0.62(−0.82-−0.43) | 2.19 | −2.14(−2.27-−2) | 69.8 | −0.34(−0.68-0) |  |
| Madagascar | 77.56 | −0.92(−1.08-−0.76) | 62.68 | −1.07(−1.17-−0.96) | 136.18 | 0.25(0.19-0.31) |  |
| Malawi | 109.94 | 0.51(0.2-0.83) | 33.48 | −1.47(−1.65-−1.3) | 151.97 | 1.15(1-1.3) |  |
| Malaysia | 184.9 | −0.45(−0.62-−0.28) | 116.81 | −1.3(−1.45-−1.14) | 416.3 | 1.6(1.42-1.78) |  |
| Maldives | 72.23 | −2.64(−2.91-−2.37) | 45.6 | −3.41(−3.72-−3.09) | 455.25 | 1.5(1.29-1.71) |  |
| Mali | 108.53 | 0.06(0-0.11) | 63.44 | −0.74(−0.84-−0.64) | 166.98 | 0.84(0.79-0.89) |  |
| Malta | 92.2 | −0.18(−0.25-−0.12) | 26.21 | −1.72(−1.78-−1.66) | 168.56 | 0.9(0.75-1.05) |  |
| Marshall Islands | 96.71 | −0.31(−0.36-−0.25) | 72.74 | −0.6(−0.68-−0.52) | 155.9 | 0.69(0.62-0.76) |  |
| Mauritania | 96.06 | 0.31(0.1-0.53) | 21.97 | −1.37(−1.64-−1.1) | 117.99 | 0.36(0.17-0.55) |  |
| Mauritius | 92.94 | −0.89(−1.03-−0.76) | 29.41 | −2.19(−2.32-−2.07) | 361.55 | 2.21(2.05-2.38) |  |
| Mexico | 118.3 | −1.01(−1.14-−0.88) | 116.37 | −1.09(−1.19-−0.98) | 458.78 | 2.5(2.41-2.59) |  |
| Micronesia  (Federated States of) | 39.88 | −0.47(−0.52-−0.42) | 25.54 | −0.59(−0.68-−0.51) | 99.81 | 0.92(0.88-0.97) |  |
| Monaco | 63.53 | 0.92(0.76-1.09) | −1.94 | −1(−1.17-−0.82) | 83.71 | 1.29(1.14-1.44) |  |
| Mongolia | 84.01 | −0.81(−1.04-−0.58) | 51.35 | −1.95(−2.18-−1.71) | 177.5 | 0.37(0.23-0.51) |  |
| Montenegro | 75.87 | 0.63(0.48-0.78) | 44.14 | −0.3(−0.47-−0.14) | 124.57 | 1.69(1.53-1.86) |  |
| Morocco | 152.71 | 0.26(0.1-0.42) | 92.14 | −0.61(−0.76-−0.47) | 275.96 | 1.62(1.44-1.8) |  |
| Mozambique | 131.99 | 1.15(1.02-1.28) | 90.13 | 0.42(0.3-0.54) | 238.46 | 2.44(2.3-2.59) |  |
| Myanmar | 30.39 | −1.54(−1.61-−1.48) | −6.53 | −2.66(−2.76-−2.56) | 226.14 | 1.95(1.86-2.03) |  |
| Namibia | 114 | 0.07(−0.2-0.34) | 65.05 | −0.7(−0.9-−0.49) | 236.43 | 2.01(1.94-2.08) |  |
| Nauru | 0.47 | −0.74(−1.01-−0.46) | 2.05 | −0.53(−0.8-−0.25) | 22.52 | −0.09(−0.41-0.23) |  |
| Nepal | 111.98 | −0.13(−0.45-0.2) | 94.38 | −0.33(−0.58-−0.08) | 278.02 | 1.78(1.55-2.01) |  |
| Netherlands | 227.85 | 2.41(2.07-2.76) | 26.93 | −1.03(−1.14-−0.92) | 124.85 | 1.12(1.01-1.24) |  |
| New Zealand | 112.36 | 0.1(0.02-0.18) | 37 | −1.38(−1.52-−1.25) | 90.71 | −0.32(−0.39-−0.25) |  |
| Nicaragua | 202.81 | −0.28(−0.5-−0.05) | 183.47 | −0.2(−0.42-0.03) | 545.52 | 3.24(3.09-3.38) |  |
| Niger | 206.85 | 0.77(0.6-0.93) | 119.81 | −0.37(−0.56-−0.18) | 193.16 | 0.31(0.2-0.42) |  |
| Nigeria | 97.91 | 0.3(0.14-0.46) | 61.7 | −0.29(−0.41-−0.16) | 180.39 | 1.65(1.44-1.85) |  |
| Niue | −2.82 | −0.32(−0.43-−0.2) | −17.16 | −0.74(−0.82-−0.66) | 39.31 | 1.22(1.16-1.28) |  |
| North Macedonia | 77.86 | 0.11(−0.05-0.28) | 25.5 | −1.35(−1.46-−1.23) | 227.06 | 2.51(2.28-2.75) |  |
| Northern Mariana Islands | 444.97 | 2.88(2.48-3.28) | 67.3 | −1.45(−1.67-−1.23) | 191.95 | 0.22(0.12-0.31) |  |
| Norway | 69.5 | 0.82(0.65-0.99) | −18.09 | −1.82(−1.91-−1.72) | 83.53 | 1.12(0.87-1.37) |  |
| Oman | 158.63 | 0.36(0.1-0.62) | 64.85 | −1.04(−1.27-−0.82) | 392.57 | 2.39(2.03-2.75) |  |
| Pakistan | 126.65 | 0.26(0.06-0.47) | 95.93 | −0.16(−0.39-0.08) | 241.73 | 1.77(1.59-1.96) |  |
| Palau | 138.13 | 0.06(−0.05-0.18) | 88.77 | −0.34(−0.41-−0.28) | 163.75 | 0.62(0.59-0.66) |  |
| Palestine | 119.42 | −0.99(−1.12-−0.85) | 82.35 | −1.45(−1.66-−1.24) | 346.95 | 1.7(1.39-2.01) |  |
| Panama | 170.37 | 0.2(−0.02-0.43) | 105.4 | −1.22(−1.38-−1.06) | 363.29 | 1.81(1.73-1.88) |  |
| Papua New Guinea | 153.57 | −0.05(−0.1-0.01) | 154.04 | −0.04(−0.12-0.04) | 221.69 | 0.64(0.59-0.69) |  |
| Paraguay | 213.53 | 0.53(0.37-0.7) | 123.77 | −1.02(−1.41-−0.62) | 535.12 | 3.4(3.26-3.54) |  |
| Peru | 104.45 | −0.97(−1.1-−0.83) | 117.79 | −0.68(−0.87-−0.49) | 509.85 | 3.38(3.14-3.62) |  |
| Philippines | 122.31 | −0.77(−0.94-−0.6) | 62.89 | −1.69(−1.86-−1.52) | 232.08 | 0.75(0.5-1.01) |  |
| Poland | 39.37 | −0.5(−0.58-−0.41) | −18.63 | −2.3(−2.38-−2.21) | 150.1 | 1.7(1.53-1.87) |  |
| Portugal | 23.86 | −0.63(−0.8-−0.45) | −5.41 | −1.85(−1.96-−1.73) | 160.84 | 1.68(1.37-2) |  |
| Puerto Rico | −18.86 | −3.3(−3.53-−3.07) | 9.33 | −2.18(−2.29-−2.07) | 230.14 | 2.15(1.85-2.45) |  |
| Qatar | 546.61 | 0.01(−0.25-0.28) | 438.91 | −0.64(−0.92-−0.35) | 1365.26 | 2.99(2.6-3.38) |  |
| Republic of Korea | 139.96 | −1.39(−1.65-−1.14) | 58.33 | −2.26(−2.6-−1.93) | 612.18 | 2.93(2.49-3.37) |  |
| Republic of Moldova | −7.5 | −0.89(−1.36-−0.42) | −32.56 | −1.48(−1.87-−1.09) | 62.13 | 1.67(1.18-2.17) |  |
| Romania | 100.82 | 1.61(1.34-1.87) | 4.18 | −0.79(−0.93-−0.65) | 182.05 | 2.76(2.62-2.9) |  |
| Russian Federation | −6.12 | −1.36(−1.6-−1.11) | −29.01 | −2.15(−2.37-−1.93) | 81.87 | 1.34(1.17-1.52) |  |
| Rwanda | 23.32 | −2.53(−2.84-−2.23) | 5.78 | −3(−3.32-−2.68) | 129.2 | 0.07(−0.17-0.32) |  |
| Saint Kitts and Nevis | 66.07 | −0.83(−1.1-−0.56) | 7.44 | −1.94(−2.15-−1.73) | 137.45 | 0.84(0.71-0.96) |  |
| Saint Lucia | 115.51 | −0.71(−0.98-−0.44) | 55.42 | −1.93(−2.22-−1.63) | 226.89 | 0.68(0.49-0.88) |  |
| Saint Vincent and  the Grenadines | 104.53 | 0.19(−0.1-0.49) | 33.81 | −1.31(−1.54-−1.08) | 158.72 | 1.06(0.94-1.18) |  |
| Samoa | 34.72 | −0.96(−1.04-−0.89) | 33.71 | −0.77(−0.82-−0.72) | 88.7 | 0.26(0.2-0.32) |  |
| San Marino | 96.47 | 0.37(0.27-0.46) | 58.27 | −0.68(−0.81-−0.55) | 123.2 | 0.81(0.74-0.89) |  |
| Sao Tome and Principe | 162.66 | 1.97(1.87-2.07) | 60.12 | −0.05(−0.15-0.06) | 185.78 | 1.95(1.87-2.04) |  |
| Saudi Arabia | 271.94 | 0.15(−0.09-0.39) | 149.25 | −1.14(−1.24-−1.04) | 852.43 | 3.54(3.42-3.67) |  |
| Senegal | 187.65 | 1.29(0.99-1.59) | 81.54 | −0.43(−0.69-−0.17) | 176.6 | 0.77(0.59-0.95) |  |
| Serbia | 33.76 | 0.22(0.06-0.38) | 1.62 | −1.18(−1.35-−1.01) | 135.43 | 2.28(2.12-2.44) |  |
| Seychelles | 110.34 | −0.15(−0.28-−0.02) | 31.09 | −1.72(−1.87-−1.58) | 294.8 | 2.01(1.76-2.26) |  |
| Sierra Leone | 142.51 | 1.38(1.16-1.6) | 54.78 | −0.18(−0.39-0.02) | 125.53 | 0.79(0.62-0.97) |  |
| Singapore | 105.39 | −1.99(−2.23-−1.75) | 66.03 | −2.6(−2.72-−2.48) | 269.46 | 0.04(−0.09-0.17) |  |
| Slovakia | 42.95 | −0.41(−0.6-−0.23) | −0.96 | −1.43(−1.67-−1.19) | 147.57 | 1.67(1.51-1.84) |  |
| Slovenia | 35.57 | −0.93(−1.05-−0.81) | 6.62 | −1.61(−1.69-−1.54) | 132.73 | 1.19(0.93-1.44) |  |
| Solomon Islands | 118.29 | −0.21(−0.3-−0.12) | 121.37 | −0.15(−0.2-−0.09) | 208.45 | 0.85(0.72-0.98) |  |
| Somalia | 90.24 | −0.99(−1.06-−0.92) | 100.64 | −0.89(−0.92-−0.85) | 151.14 | 0.05(−0.05-0.15) |  |
| South Africa | 49.53 | −1.8(−2.38-−1.21) | 38.07 | −1.63(−2.03-−1.24) | 151.59 | 0.74(0.6-0.88) |  |
| South Sudan | 22.33 | −1.18(−1.24-−1.13) | 15.59 | −1.19(−1.23-−1.14) | 61.46 | 0.04(−0.04-0.13) |  |
| Spain | 35.81 | −0.89(−0.95-−0.83) | 19.9 | −1.13(−1.32-−0.93) | 160.48 | 1.57(1.45-1.69) |  |
| Sri Lanka | 140.13 | 0.56(0.2-0.92) | 57.04 | −1.23(−1.37-−1.08) | 355.07 | 2.68(2.57-2.8) |  |
| Sudan | 123.39 | 0.37(0.25-0.48) | 77.96 | −0.33(−0.39-−0.27) | 239.41 | 1.74(1.62-1.86) |  |
| Suriname | 140.03 | 0.05(−0.24-0.34) | 70.62 | −1.26(−1.56-−0.96) | 256.64 | 1.69(1.53-1.85) |  |
| Sweden | 57.03 | 0.41(0.28-0.55) | −31.42 | −2.53(−2.65-−2.42) | 59.13 | 0.42(0.22-0.62) |  |
| Switzerland | 44.13 | −0.34(−0.44-−0.24) | −14.53 | −2.18(−2.42-−1.94) | 89.27 | 0.18(−0.12-0.48) |  |
| Syrian Arab Republic | 144.54 | −0.06(−0.26-0.15) | 90.43 | −0.57(−0.81-−0.34) | 215.87 | 1.3(1.13-1.48) |  |
| Taiwan  (Province of China) | 390.8 | 3.18(2.88-3.48) | 111.8 | −0.5(−0.67-−0.33) | 525.34 | 3.56(3.25-3.87) |  |
| Tajikistan | 2.39 | −1.72(−2.15-−1.28) | 31.34 | −0.49(−0.78-−0.2) | 71.2 | 0.51(0.07-0.94) |  |
| Thailand | 141.17 | −1.07(−1.31-−0.82) | 60.62 | −2.3(−2.53-−2.07) | 326.13 | 1.14(0.85-1.44) |  |
| Timor-Leste | 146.68 | −0.38(−0.66-−0.1) | 90.42 | −1.21(−1.48-−0.93) | 398.64 | 2.61(2.32-2.91) |  |
| Togo | 283.54 | 1.19(1.03-1.34) | 134.1 | −0.4(−0.53-−0.27) | 235.25 | 0.51(0.45-0.57) |  |
| Tokelau | −15.97 | −0.6(−0.63-−0.57) | −25.56 | −1(−1.02-−0.97) | 38.78 | 1.17(1.12-1.22) |  |
| Tonga | 44.99 | −0.21(−0.49-0.08) | 26.35 | −0.52(−0.7-−0.34) | 80.81 | 0.68(0.56-0.81) |  |
| Trinidad and Tobago | 47.42 | −1.95(−2.3-−1.6) | −5.83 | −3.41(−3.71-−3.11) | 164.84 | 0.66(0.55-0.77) |  |
| Tunisia | 181.55 | 0.27(0.2-0.33) | 122.74 | −0.55(−0.61-−0.49) | 344.43 | 1.87(1.81-1.93) |  |
| Turkey | 81.36 | −0.93(−1.16-−0.7) | 50.35 | −1.24(−1.87-−0.61) | 288.69 | 2.13(1.66-2.61) |  |
| Turkmenistan | −35.93 | −4.29(−5.01-−3.55) | −10.27 | −3.13(−3.5-−2.76) | 96.37 | −0.09(−0.6-0.42) |  |
| Tuvalu | 18.21 | −0.92(−0.99-−0.85) | 10.74 | −0.93(−0.98-−0.88) | 85.88 | 0.57(0.5-0.63) |  |
| Uganda | 173.42 | 0.67(0.48-0.85) | 94.32 | −0.97(−1.25-−0.69) | 321.52 | 2.12(1.91-2.32) |  |
| Ukraine | −12.75 | −1.16(−1.55-−0.77) | −40.93 | −2.68(−3.03-−2.32) | 14.4 | −0.09(−0.33-0.16) |  |
| United Arab Emirates | 1132.05 | 0.3(−0.04-0.64) | 519.22 | −1.76(−2.16-−1.37) | 1070.57 | 0.36(−0.02-0.74) |  |
| United Kingdom | 61.14 | 0.4(0.23-0.56) | −16.99 | −1.93(−2.08-−1.78) | 51.36 | 0.28(0.24-0.32) |  |
| United Republic of Tanzania | 81.9 | −0.86(−0.92-−0.8) | 68.61 | −1.04(−1.07-−1) | 199.52 | 0.98(0.88-1.08) |  |
| United States of America | 93.45 | 0.14(0-0.29) | 31.64 | −1.05(−1.14-−0.96) | 52.73 | −0.6(−0.71-−0.49) |  |
| United States Virgin Islands | 155.14 | 1.18(0.88-1.47) | 103.42 | 0.12(−0.07-0.31) | 273.97 | 2.31(2.02-2.6) |  |
| Uruguay | −7.92 | −1.54(−1.68-−1.41) | −7.71 | −1.34(−1.41-−1.28) | 67.18 | 0.68(0.58-0.79) |  |
| Uzbekistan | −23.01 | −3.97(−4.41-−3.52) | 17.4 | −1.96(−2.11-−1.81) | 128.56 | 0.87(0.71-1.03) |  |
| Vanuatu | 163.95 | −0.23(−0.37-−0.09) | 146.81 | −0.42(−0.54-−0.3) | 229.2 | 0.6(0.44-0.75) |  |
| Venezuela  (Bolivarian Republic of) | 152.95 | −0.75(−0.9-−0.6) | 127.97 | −1.26(−1.43-−1.09) | 446.2 | 1.89(1.69-2.09) |  |
| Viet Nam | 214.09 | 1.27(1.11-1.44) | 49.56 | −1.98(−2.14-−1.82) | 603.72 | 4.29(4.14-4.44) |  |
| Yemen | 159.75 | −0.12(−0.15-−0.09) | 153.75 | −0.24(−0.3-−0.18) | 270.75 | 1.39(1.27-1.52) |  |
| Zambia | 91.11 | −1.12(−1.34-−0.89) | 76.84 | −1.38(−1.63-−1.13) | 231.71 | 1.04(0.97-1.1) |  |
| Zimbabwe | 81.44 | 0.09(−0.16-0.35) | 85.53 | 0.66(0.39-0.94) | 122.03 | 0.66(0.44-0.89) |  |

YLDs: years lived with disability; EAPCs: estimated annual percentage changes; ASR, age-standardized rate; CI, confidence interval; UI: uncertainty interval. Percentage change in number was calculated based on the crew data.
